# Supplementary material for: Deciphering an AgRP-serotoninergic neural circuit in distinct control of energy metabolism from feeding
Source: Nat Commun. 2021 Jun 10;12:3525. doi: 10.1038/s41467-021-23846-x (PMC8192783; doi:10.1038/s41467-021-23846-x)
Supplement: Supplementary file 1 — Supplementary Information [file 41467_2021_23846_MOESM1_ESM.pdf]

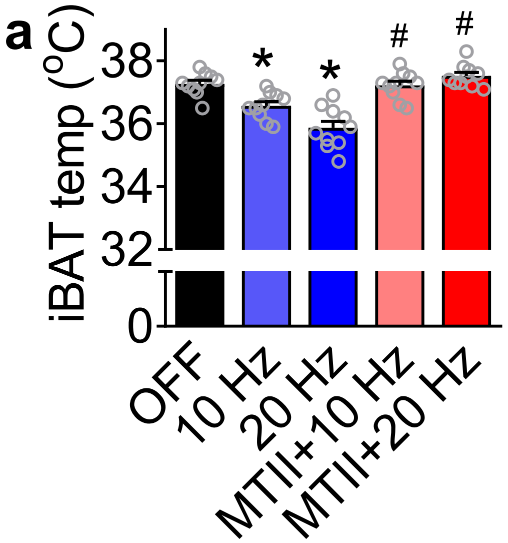

Supplementary Fig. 1. The effects of MTII on the hypothermia induced by optogenetic activation of AgRP→dlDRN circuit. a iBAT (intrascapular brown adipose tissue) temperature after 10 Hz and 20 Hz optogenetic activation of the AgRP→dlDRN circuit with or without a pretreatment of 4 ng MTII (melanotan II) into the dlDRN. (n = 10 per group; **P* was calculated between OFF and photostimulation (10 Hz or 20 Hz), $^{\#}P$ was calculated between 10 Hz and MTII+10 Hz, or between 20 Hz and MTII+20 Hz; F = 21.52, **P* =0.0109 at 10 Hz, P <0.0001 at 20 Hz, $^{\#}P$ =0.0211 at MTII+10 Hz, P <0.0001 at MTII+20 Hz; one-way ANOVA followed by Tukey post hoc test). Error bars represent mean ± s.e.m.

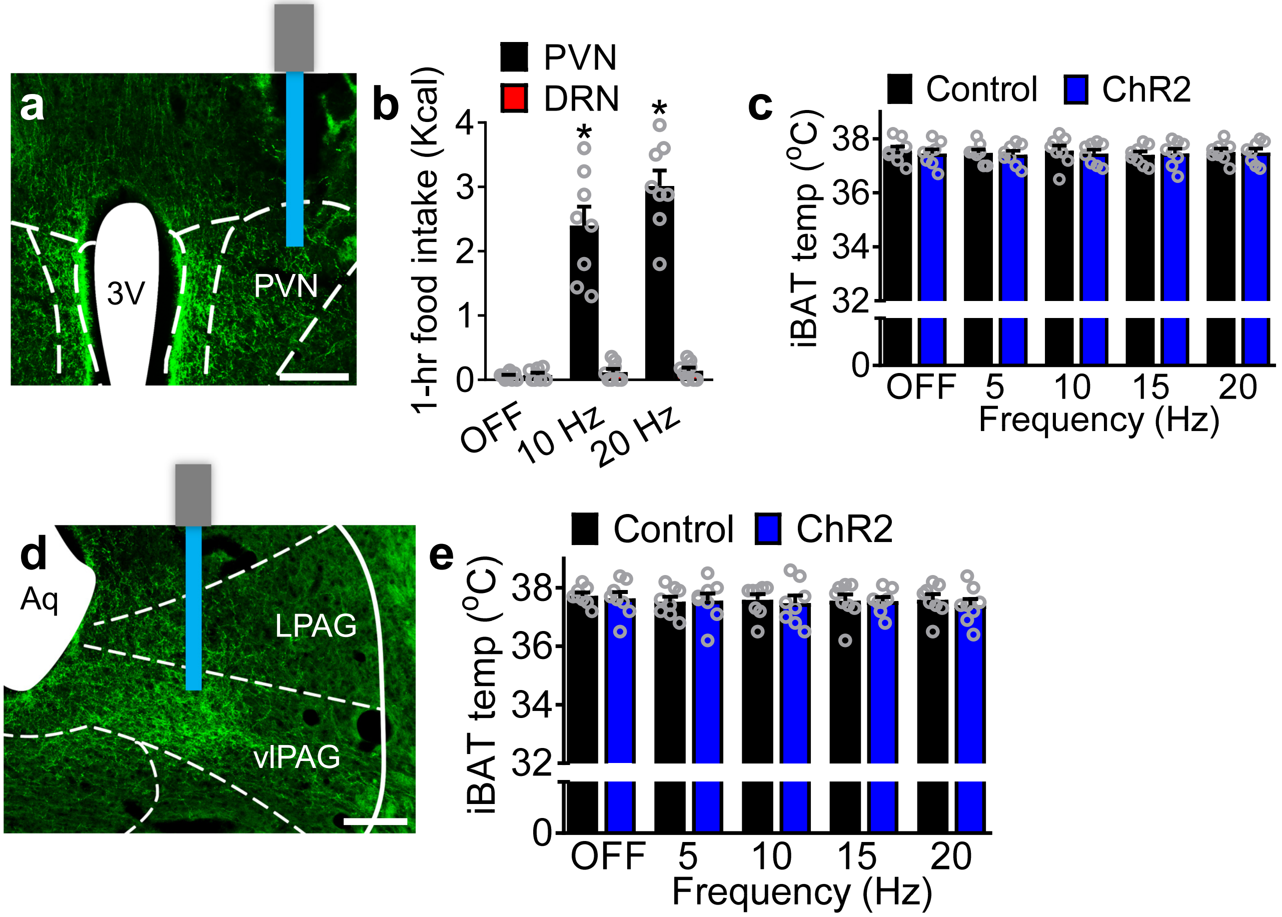

Supplementary Fig. 2. Optogenetic activation of the downstream targets of AgRP neurons in PVN and vlPAG. a Representative image showing the placement of an optical fiber in the PVN in *Agrp*^{Cre}::Ai32 mice. Scale bar, 200 µm. **b** One-hour food intake by ad lib-fed *Agrp*^{Cre}::Ai32 mice under sham, 10 Hz, and 20 Hz photostimulation of the AgRP→PVN or AgRP→dlDRN circuit. (n = 8 per group; F = 165.5, *P was calculated between OFF and 10 Hz or 20 Hz; *P < 0.0001 at10 Hz and 20 Hz; two-way ANOVA followed by Bonferroni post hoc test). **c** iBAT temperature after photostimulation of the AgRP→PVN circuit at 5 Hz, 10 Hz, 15 Hz, and 20 Hz. (n = 8 per group; Control vs ChR2; P = 0.989 at Frequency 0, P = 0.999 at Frequency 5, P = 0.989 at Frequency 10, P = 0.999 at Frequency 15, P > 0.999 at Frequency 20; one-way ANOVA followed by Tukey post hoc test). **d** Representative image showing the placement of optical fibers in the vlPAG in *Agrp*^{Cre}::Ai32 mice. Scale bar, 200 µm. **e** iBAT temperature after photostimulation of the AgRP→vlPAG neural circuit at 5 Hz, 10 Hz, 15 Hz, and 20 Hz. (n = 8 per group; Control vs ChR2; P = 0.999 at Frequency 0, P > 0.999 at Frequency 5, P = 0.997 at Frequency 10, P > 0.999 at Frequency 15, P = 0.973 at Frequency 20; one-way ANOVA followed by Tukey post hoc test). Error bars represent mean ± s.e.m.

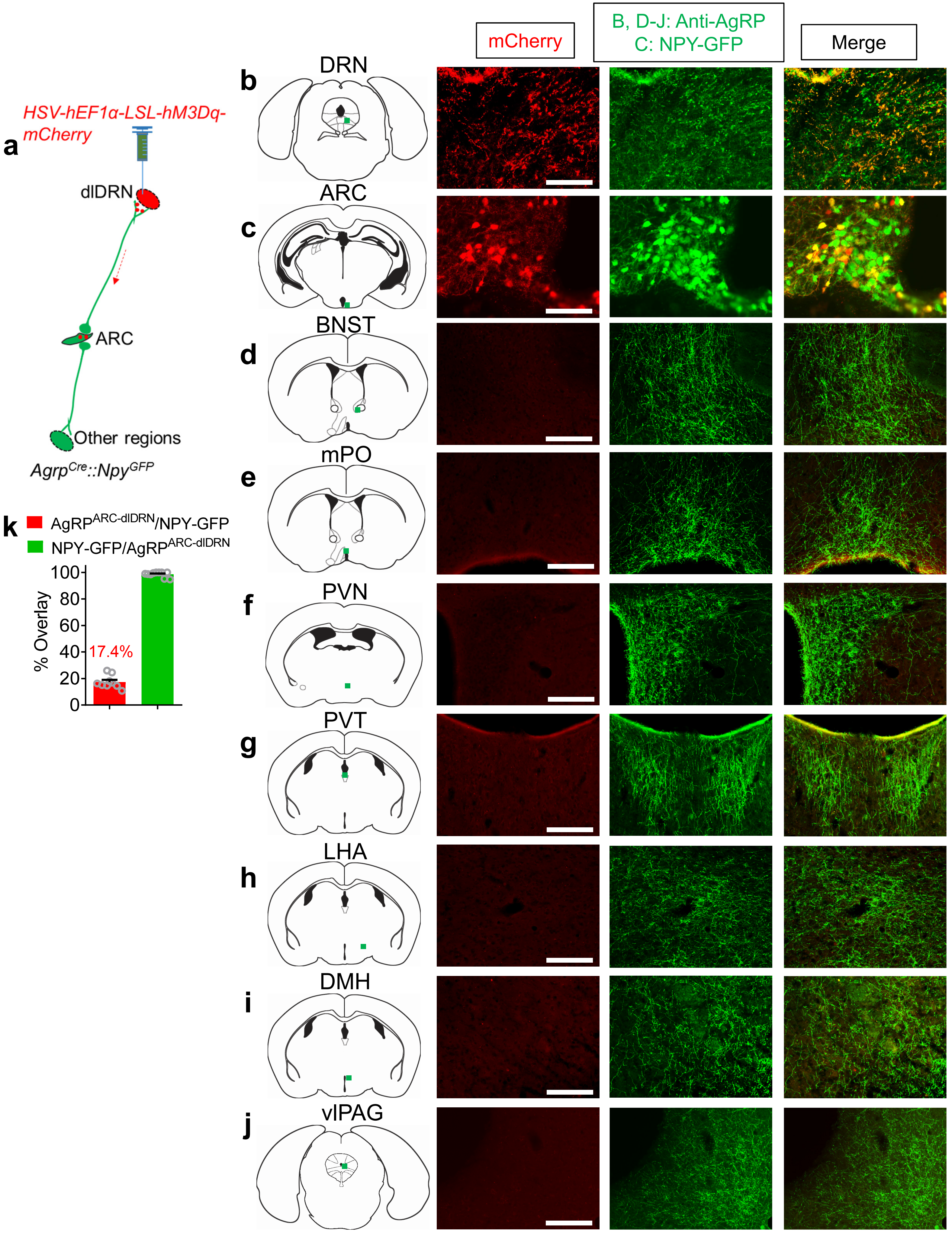
a
HSV-hEF1α-LSL-hM3Dq-mCherry
dlDRN
ARC
Other regions
AgrpCre::NpyGFP
k
AgRPARC-dlDRN/NPY-GFP
NPY-GFP/AgRPARC-dlDRN
% Overlay
17.4%
100
80
60
40
20
0
mCherry
B, D-J: Anti-AgRP
C: NPY-GFP
Merge
b
DRN
c
ARC
d
BNST
e
mPO
f
PVN
g
PVT
h
LHA
i
DMH
j
vlPAG

Supplementary Fig. 3. AgRP$^{ARC\rightarrow dlDRN}$ neurons did not collaterally project to other AgRP downstream targets. a Diagram shows retrograde-targeting AgRP$^{ARC\rightarrow dlDRN}$ neurons by injection of *HSV-hEF1α-LSL-hM3Dq-mCherry* into the dlDRN of *Agrp^{Cre}::Npy^{GFP}* mice. The signals were examined 4 weeks later. **b-j** mCherry fluorescence was observed in the DRN (**b**) and ARC (**c**) but not in the BNST (**d**), mPO (**e**), PVN (**f**), PVT (**g**), LHA (**h**), DMH (**i**) and vlPAG (**j**). The immunostaining of AgRP was performed in the brain region showed in **b**, **d-j**. The green box in the schematics indicating the brain regions examined. Scale bars in **b-j**, 100 μm. **k** Statistical analysis of the percentage of AgRP$^{ARC\rightarrow dlDRN}$ neurons in all NPY/AgRP neurons. (n = 10 brain sections from 4 animals per group). Error bars represent mean $\pm$ s.e.m.

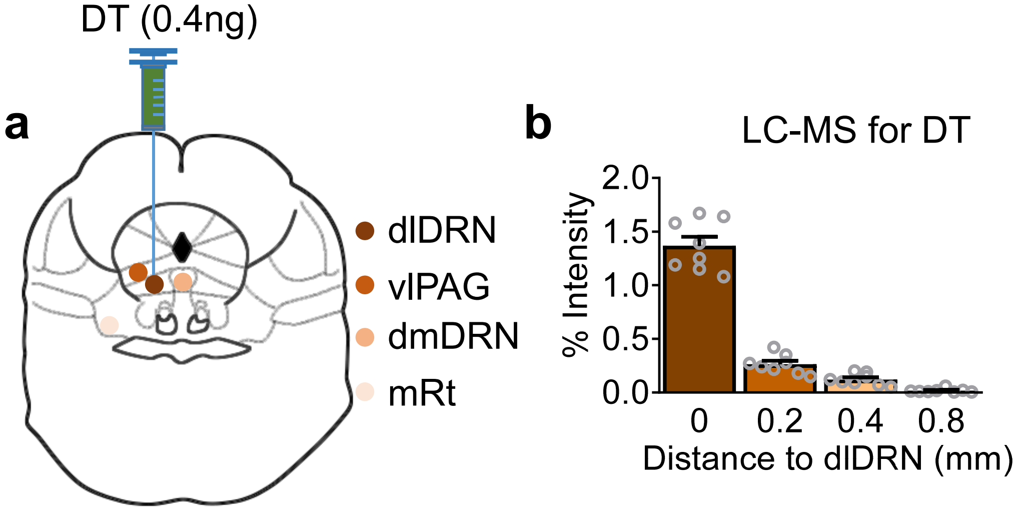

Supplementary Fig. 4. Quantification of DT diffusion in the brain tissue. a Schematic diagram showing that DT (0.4 ng) was injected into the dlDRN, and four punches of brain tissue containing dlDRN, dmDRN, vlPAG, and mRt were collected 24 hours later. **b** The quantitative results for brain regions in different distances were calculated based on the absolute LC-MS (liquid chromatography with tandem mass spectrometry) ion intensity values. For each data point with different distance, the ion intensity score was calculated by the chromatographic peak areas for DT (diphtheria toxin) against the total ion area of each sample and subsequently normalized to tissue weight. (n = 8 samples from 8 animals). Error bars represent mean ± s.e.m.

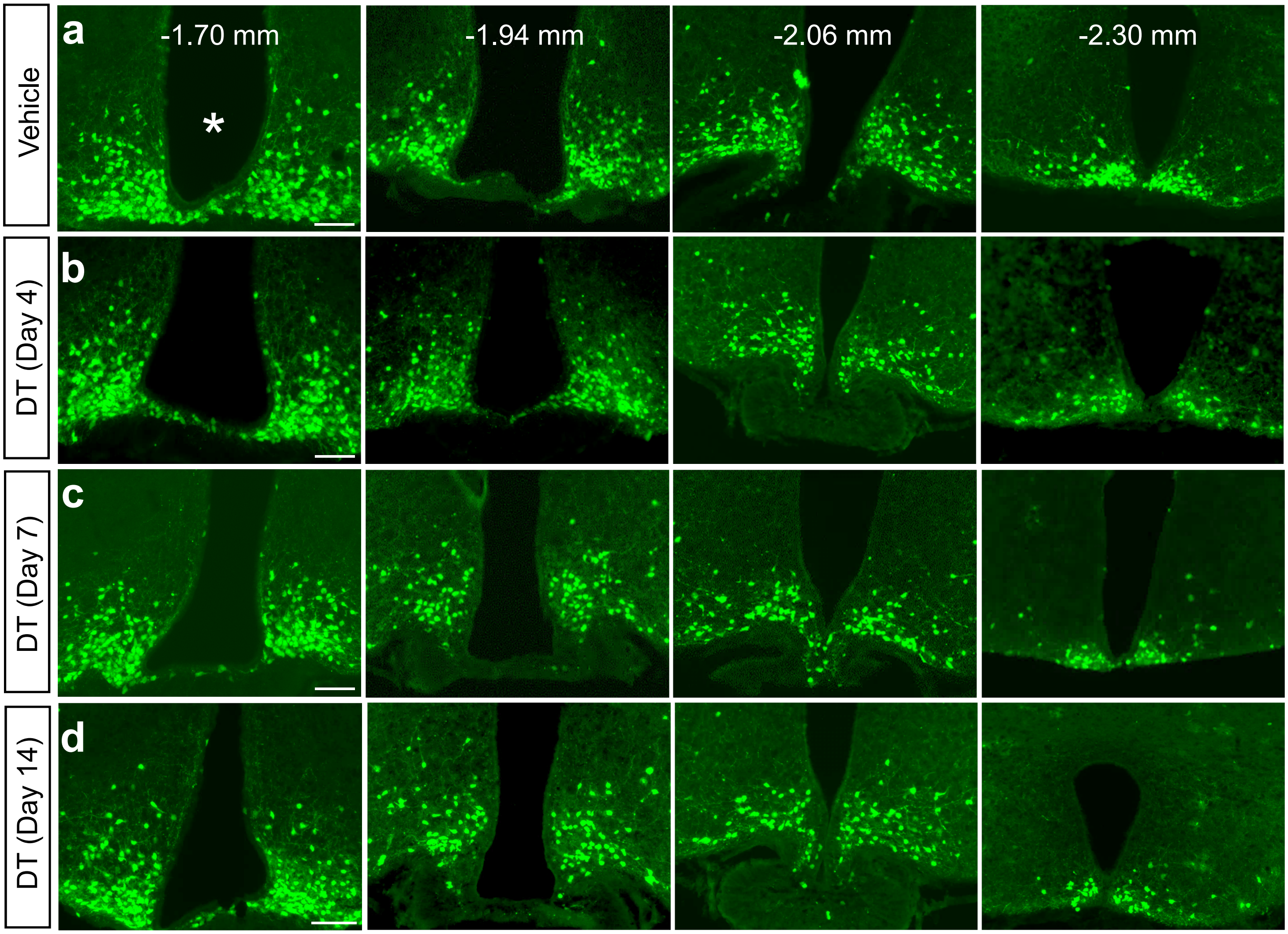

Supplementary Fig. 5. The distribution of AgRP$^{ARC \rightarrow dlDRN}$ neurons in ARC. a-d Representative images showing the expression profile of NPY/AgRP neurons on days 4, 7, and 14 after injection of DT or vehicle (0.9% NaCl) into the dlDRN of *Agrp*$^{DTR/+}$*:: Npy*^{GFP} mice. Asterisk in **a** indicates the third ventricle. Scale bars in **a-d**, 100 μm.

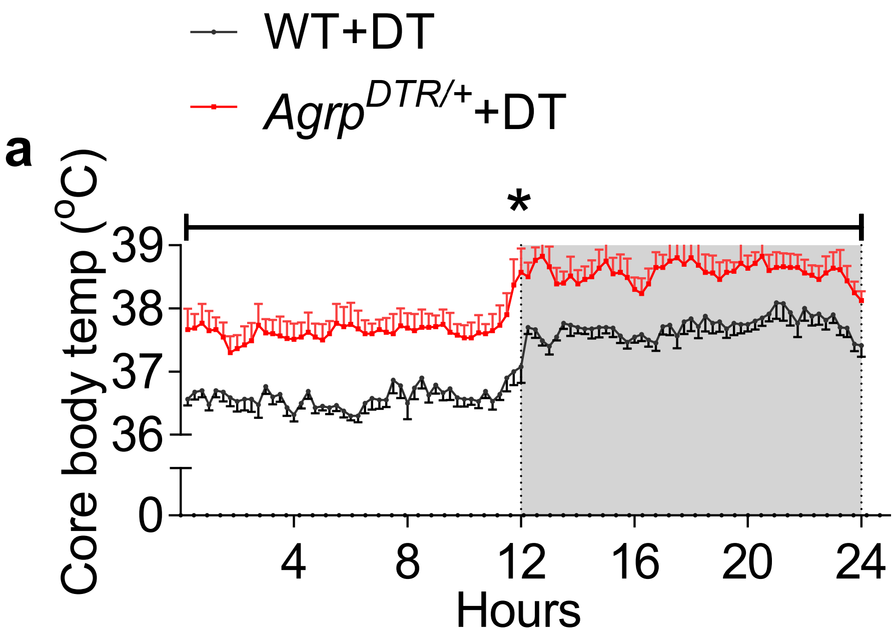

Supplementary Fig. 6. Real-time core body temperature in the ablation of AgRP$^{ARC\rightarrow dlDRN}$ neurons. a 24-hour real-time core body temperature 7 days after injection of DT into the dlDRN of either WT or *Agrp*$^{DTR/+}$ mice. (n = 8 per group; F =19.16, *P < 0.0001, *Agrp*$^{DTR/+}$+DT vs WT+DT; two-way ANOVA followed by Bonferroni post hoc test). Error bars represent mean ± s.e.m.

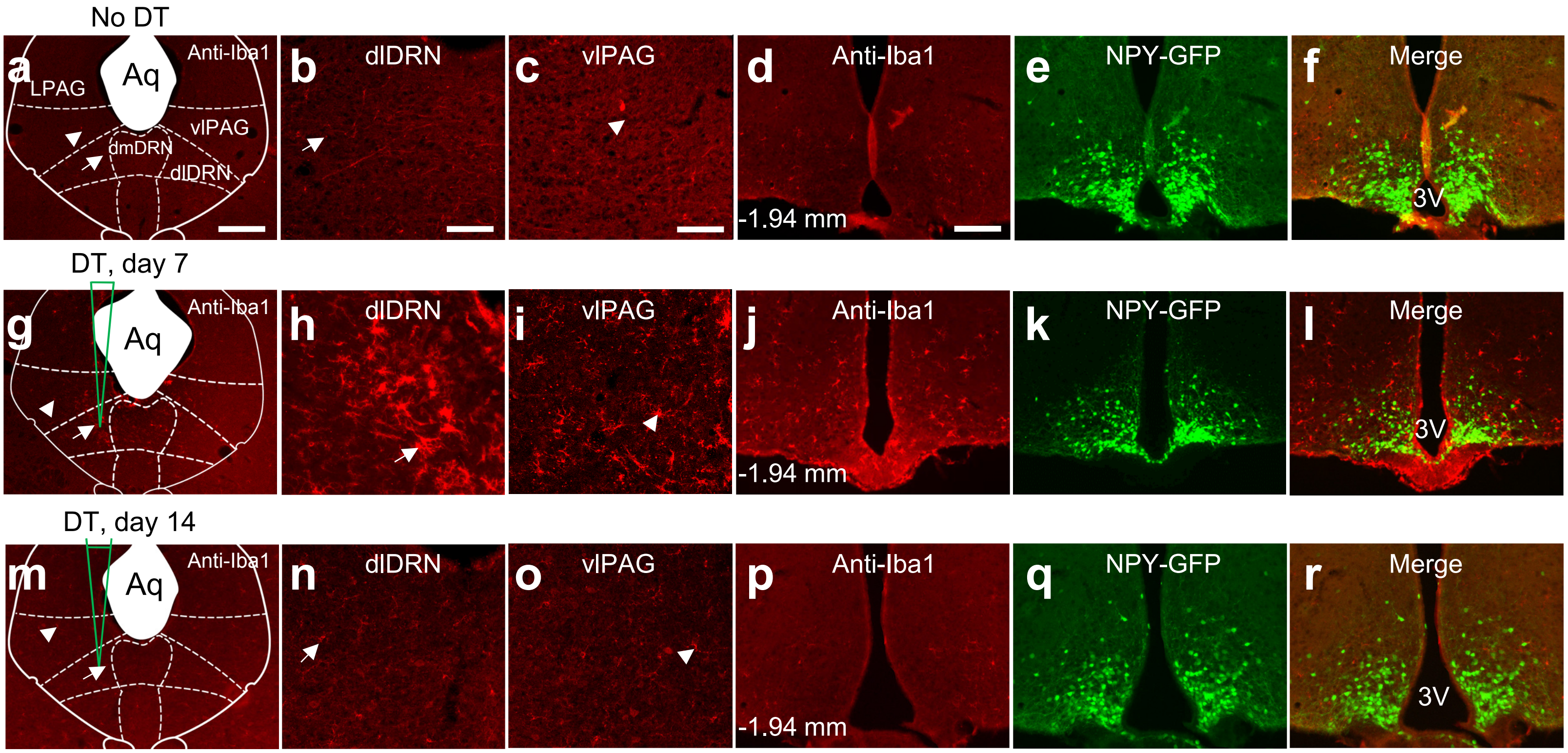

Supplementary Fig. 7. Gliosis was transiently induced and disappeared 14 days after ablation of AgRP$^{\mathbf{ARC \rightarrow dlDRN}}$ neurons. a-f Immunostaining of microglial marker Iba1 (Ionized calcium binding adaptor molecule 1) in the DRN (**a**, **b**), vlPAG (**c**) and ARC (**d-f**) of *Agrp*$^{DTR/+}$*::Npy*^{GFP} mice lacking a DT injection into the dlDRN. **g-l** Immunostaining of Iba1 in the DRN (**g**, **h**), vlPAG (**i**) and ARC (**j-l**) 7 days after DT injection into the dlDRN of *Agrp*$^{DTR/+}$*::Npy*^{GFP} mice. **m-r** Immunostaining of Iba1 in the DRN (**m**, **n**), vlPAG (**o**) and ARC (**p-r**) 14 days after DT injection into the dlDRN of *Agrp*$^{DTR/+}$*::Npy*^{GFP} mice. Arrows in **a**, **g**, **m** indicate dlDRN, and arrowheads indicate the vlPAG, with magnified views in **b** and **c**, **h** and **i**, **n** and **o**, respectively. Scale bar in **a** for **a**, **g**, **m**, 200 μm; scale bar in **b** for **b**, **h**, **n**, 50 μm; scale bar in **c** for **c**, **i**, **o**, 50 μm; scale bar in **d** for **d-f**, **j-l**, **p-r**, 100 μm.

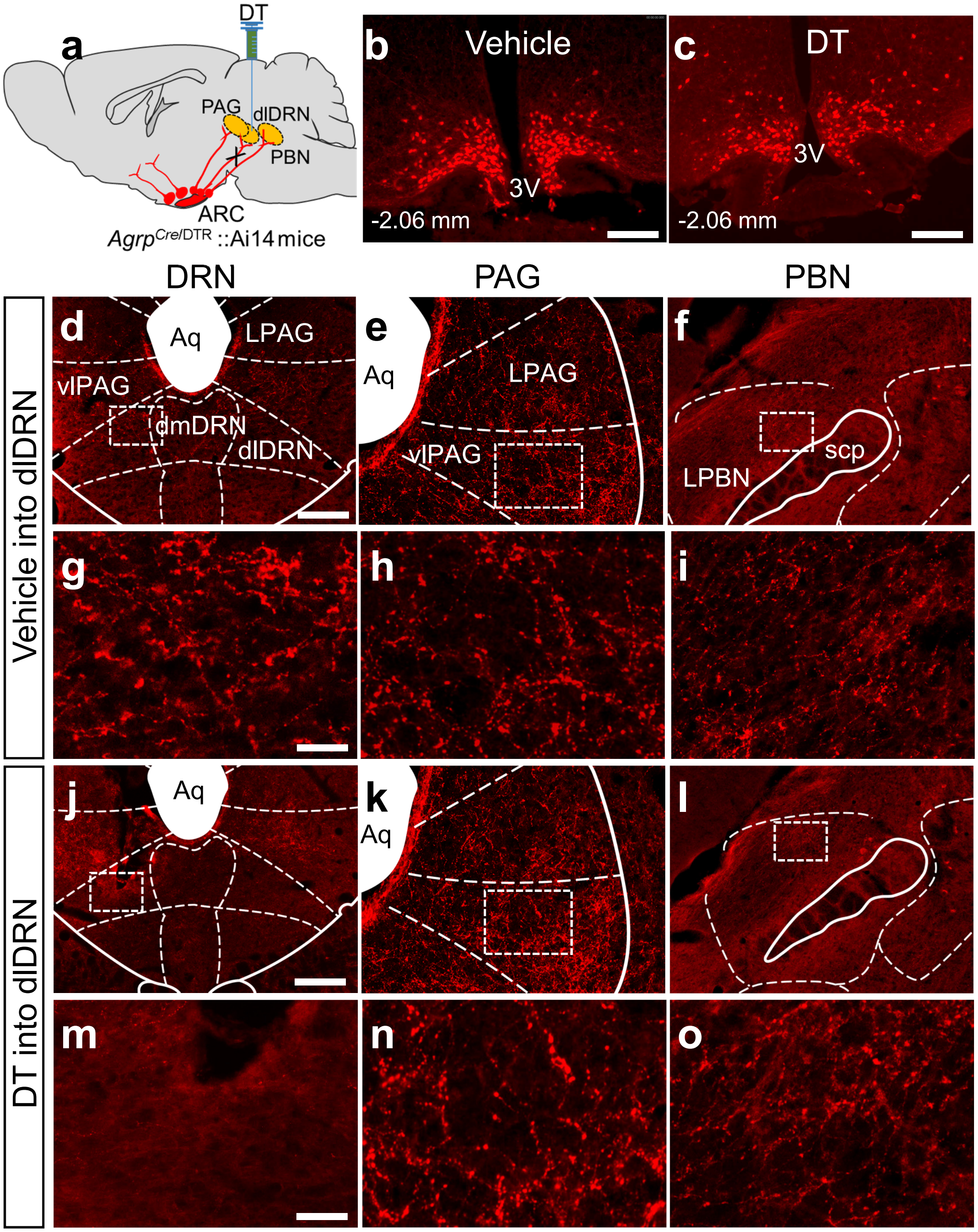

Supplementary Fig. 8. Distribution of AgRP fibers in hindbrain after ablation of AgRP$^{ARC\rightarrow dlDRN}$ neurons. **a** Diagram showing ablation of a subpopulation of AgRP neurons resulting from DT injected into the dlDRN of *Agrp$^{Cre/DTR}$*::Ai14 mice. **b-c** Representative images showing AgRP neurons in coronal sections 7 days after injection of vehicle (**b**) or DT (**c**) into the dlDRN of *Agrp$^{Cre/DTR}$*::Ai14 mice. Scale bars in **b** and **c**, 100 μm. **d-f**, **j-l** Representative images with low magnification showing the expression of AgRP fibers in the DRN, PAG and PBN of *Agrp$^{Cre/DTR}$*::Ai14 mice 7 days after treatment with either vehicle (**d-f**) or DT (0.4 ng/side) into the dlDRN (**j-l**). Scale bars in **d** for **d-f** and **j** for **j-l**, 200 μm. **g-i**, **m-o** Representative images with high magnification for **d-f** and **j-l**. Scale bars in **g** for **g-i** and **m** for **m-o**, 50 μm.

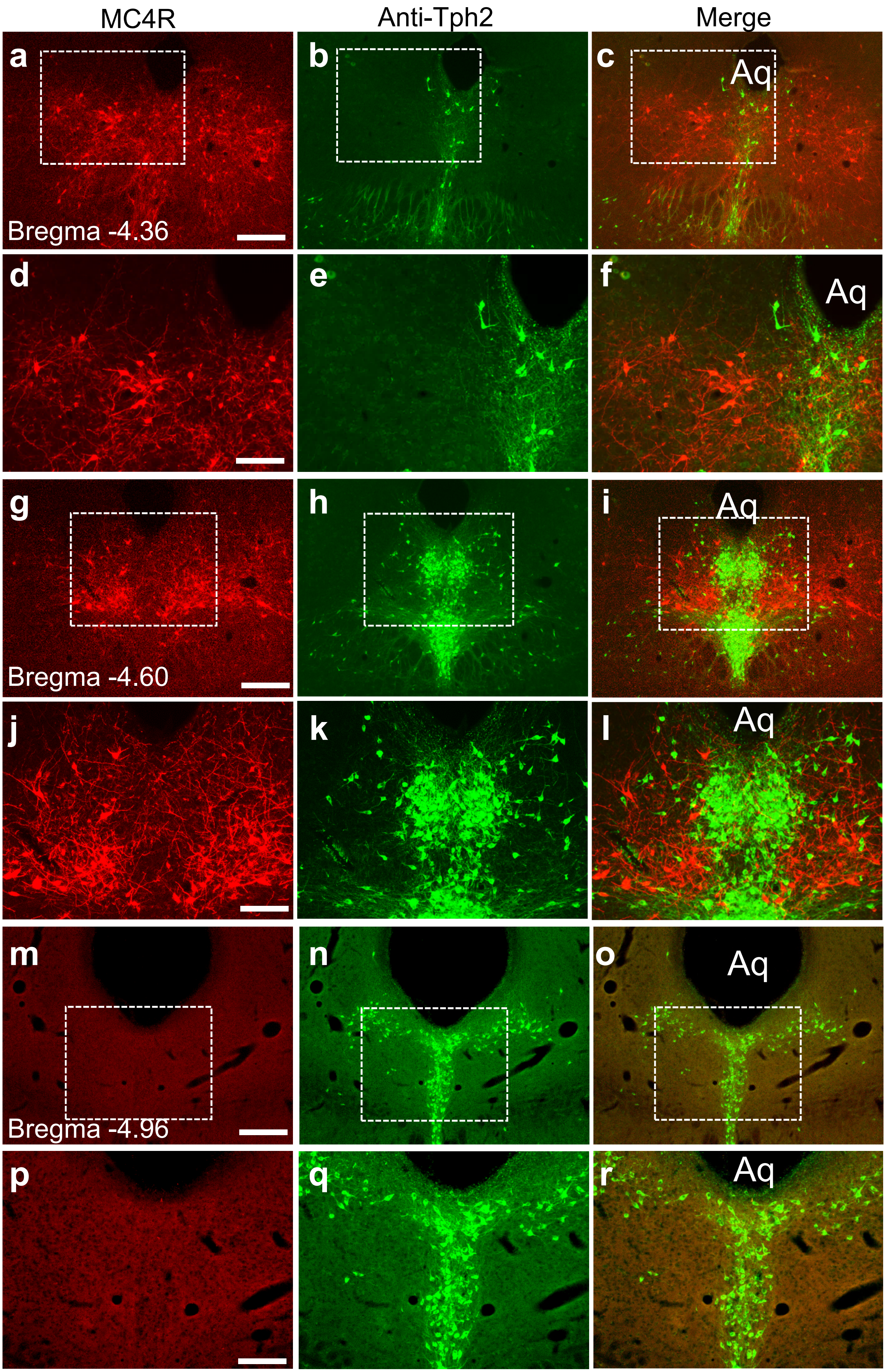

MC4R
Anti-Tph2
Merge
a
b
c
Aq
Bregma -4.36
d
e
f
Aq
g
h
i
Aq
Bregma -4.60
j
k
l
Aq
m
n
o
Aq
Bregma -4.96
p
q
r
Aq

Supplementary Fig. 9. Anatomical profiling of MC4R neurons and 5-HT neurons in the DRN. a-r Representative images showing MC4R (mCherry) and 5-HT signaling (GFP, anti-Tph2) in the dlDRN (**a-f**, Bregma -4.36 mm; **g-l**, Bregma -4.60 mm; **m-r**, Bregma -4.96 mm) of *Mc4r*^{Cre} mice with *AAV9-DIO-mCherry* injection into the dlDRN. Scale bars in **a**, **g**, **m** for **a-c**, **g-i**, **m-o**, respectively, 200 μm; scale bars in **d**, **j**, and **p** for **d-f**, **j-l**, **p-r**, respectively, 100 μm.

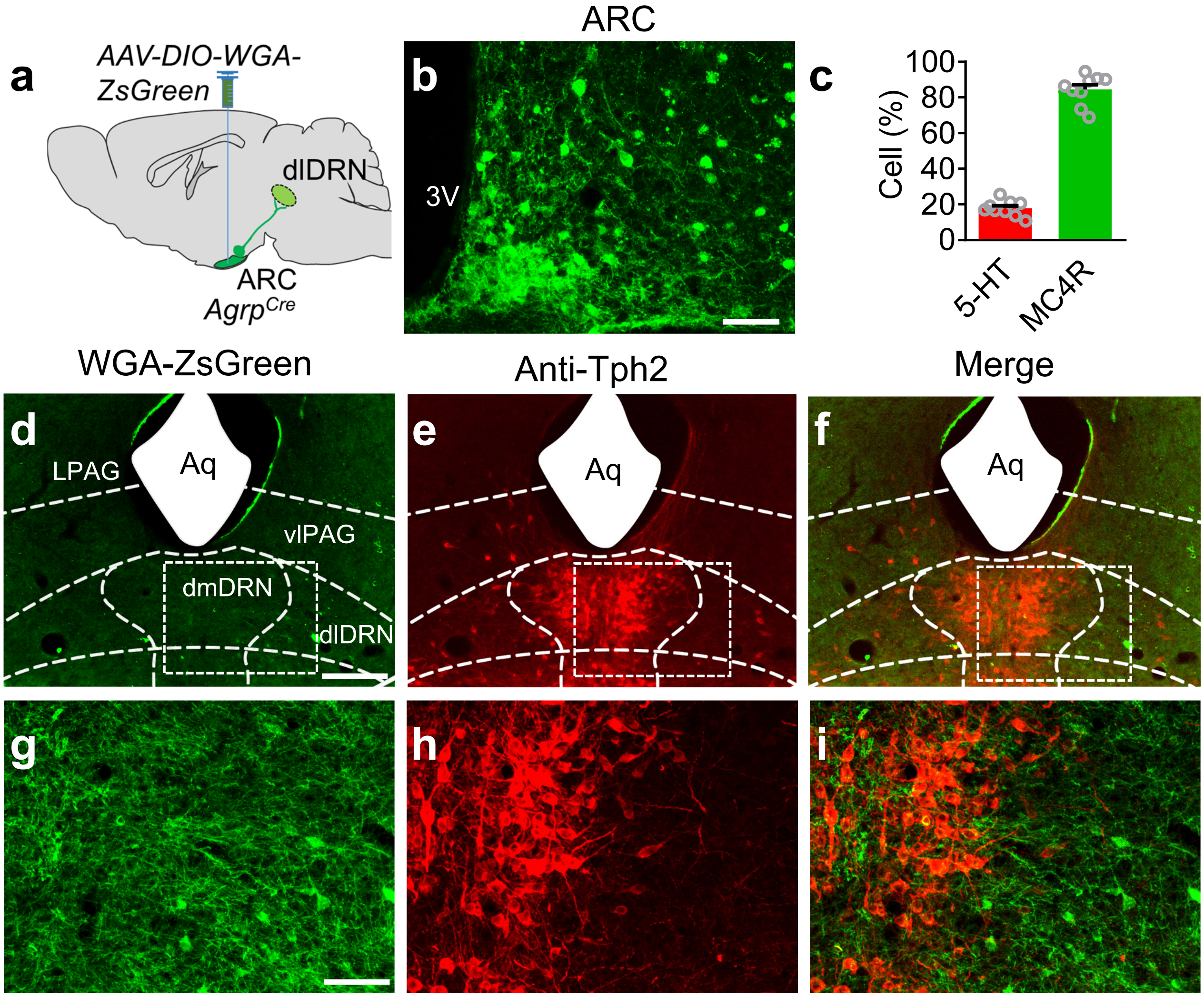

Supplementary Fig. 10. Transsynaptic tracing from AgRP neurons to the dlDRN. a Schematic illustration showing transsynaptic *AAV9-DIO-WGA-ZsGreen* injected into the ARC of *Agrp*^{Cre} mice. **b** Representative images showing WGA-ZsGreen in the ARC. Scale bar, 100 μm. **c** Statistical analysis showing the percentage of MC4R neurons and 5-HT neurons within the DRN. (n = 9 per group). **d-i** WGA-ZsGreen-labeled MC4R neurons in the dlDRN and anti-Tph2 in the dmDRN. Scale bar in **d** for **d-f**, 100 μm; scale bar in **g** for **g-i**, 50 μm. Error bars represent mean ± s.e.m.

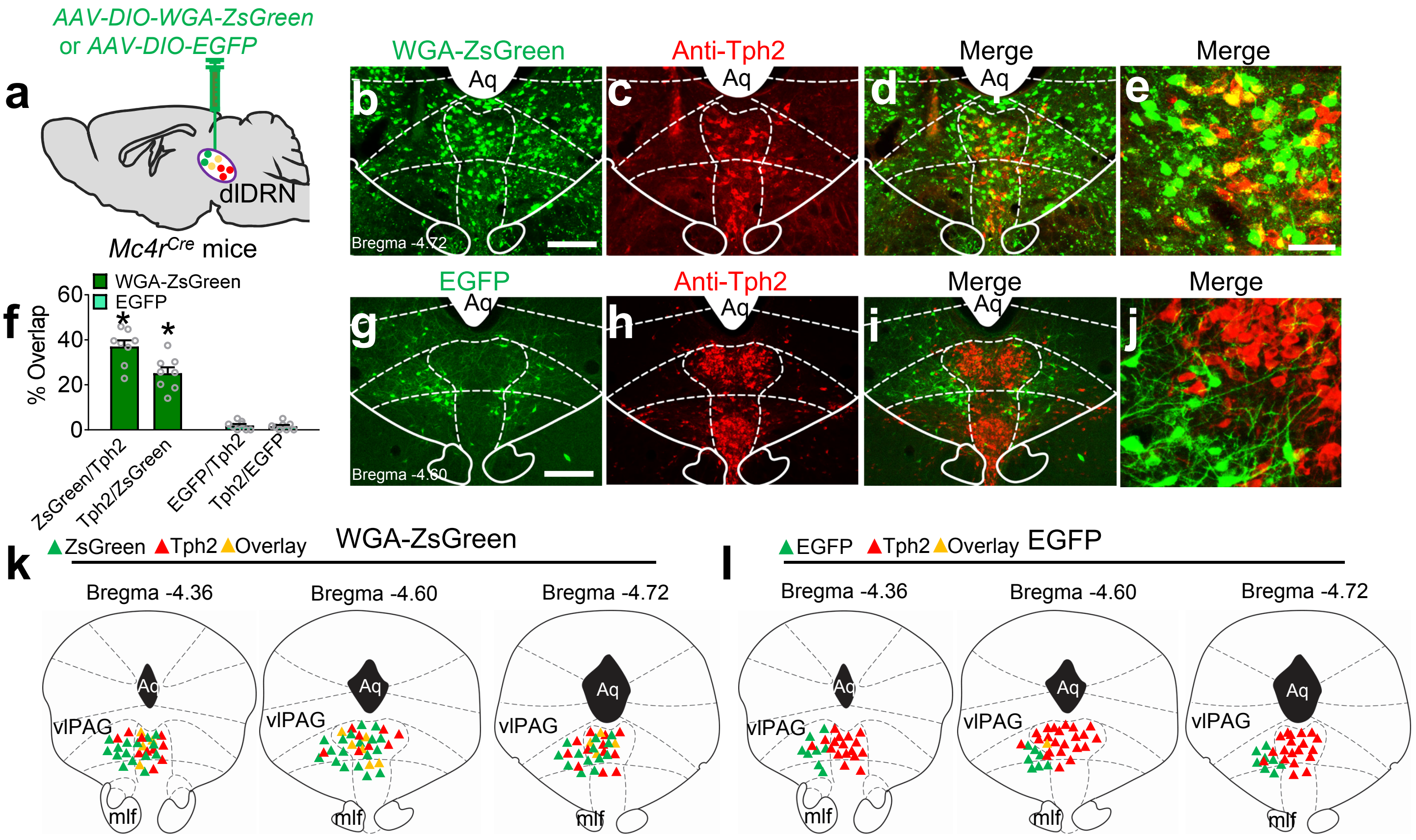

Supplementary Fig. 11. Transsynaptic tracing from MC4R^{dlDRN} neurons to 5-HT^{dmDRN} neurons. a Schematic illustration of tracing from MC4R^{dlDRN} to the 5-HT^{dmDRN} neurons. *Mc4r^{Cre}* mice were injected with the *AAV9-DIO-WGA-ZsGreen* or *AAV9-DIO-EGFP* virus into the dlDRN. **b-e** Representative images showing the expression of WGA-ZsGreen and Tph2 in the dlDRN and dmDRN. Scale bar in **b** for **b-d**, 200 μm; scale bar in **e**, 50 μm. **f** Statistical data showing the percentage of connectivity between 5-HT^{dmDRN} neurons and MC4R^{dlDRN} neurons as revealed by the WGA-ZsGreen group and the EGFP group. (n = 8 per group; *P was calculated between ZsGreen/Tph2 and EGFP/Tph2, or between Tph2/ZsGreen and Tph2/EGFP; F = 224.7, *P < 0.0001; Two-way ANOVA followed by Bonferroni post hoc test). **g-j** Representative images showing EGFP and Tph2 in the dlDRN and dmDRN. Scale bar in **g** for **g-i**, 200 μm; scale bar in **j**, 50 μm. **k-l** Schematic images showing the colocolization of WGA-ZsGreen (**k**) or EGFP (**l**) with Tph2 in various coronal sections. Error bars represent mean ± s.e.m.

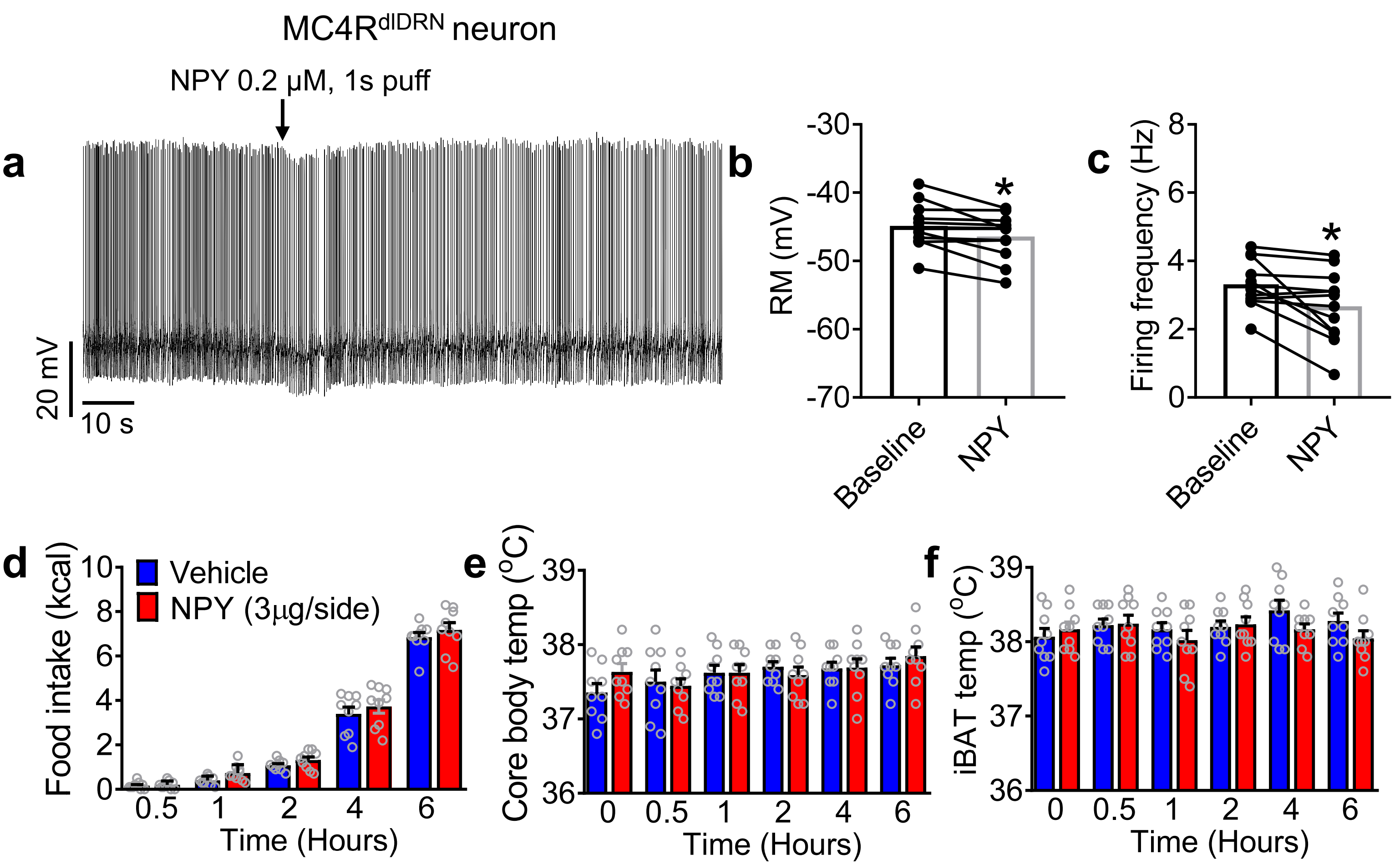

Supplementary Fig. 12. Effects of NPY on MC4R^{dlDRN} neurons. a Representative trace from patch clamp recordings of MC4R^{dlDRN} neurons treated with NPY (1s puff) in mice with injection of *AAV2-DIO-ChR2-GFP* into the dlDRN of *Mc4r^{Cre}* mice. **b-c** RM potential (**b**) and firing frequency (**c**) of MC4R^{dlDRN} neurons as showed in **a** (n = 12 neurons from 3 mice; for **b**, *P =0.0135; for **c**, *P =0.013; paired two-tailed t test). **d-f** The food intake (**d**), core body temperature (**e**) and iBAT temperature (**f**) of WT mice with infusion of vehicle or NPY into the dlDRN (n = 9 per group; all comparisons in **d-f** were made between Vehicle and NPY; for **d**: P > 0.999 at hour 0.5, P = 0.736 at hour 1, P = 0.924 at hour 2, P = 0.736 at hour 4, P = 0.736 at hour 6; for **e**: P = 0.385 at hour 0, P = 0.999 at hour 0.5, P > 0.999 at hour 1, P = 0.979 at hour 2, P > 0.999 at hour 4 , P = 0.967 at hour 6; for **f**: P = 0.985 at hour 0, P >0.999 at hour 0.5, P = 0.911 at hour 1, P > 0.999 at hour 2, P = 0.424 at hour 4 , P = 0.531 at hour 6; two-way ANOVA followed by Bonferroni post hoc test). Error bars represent mean ± s.e.m.

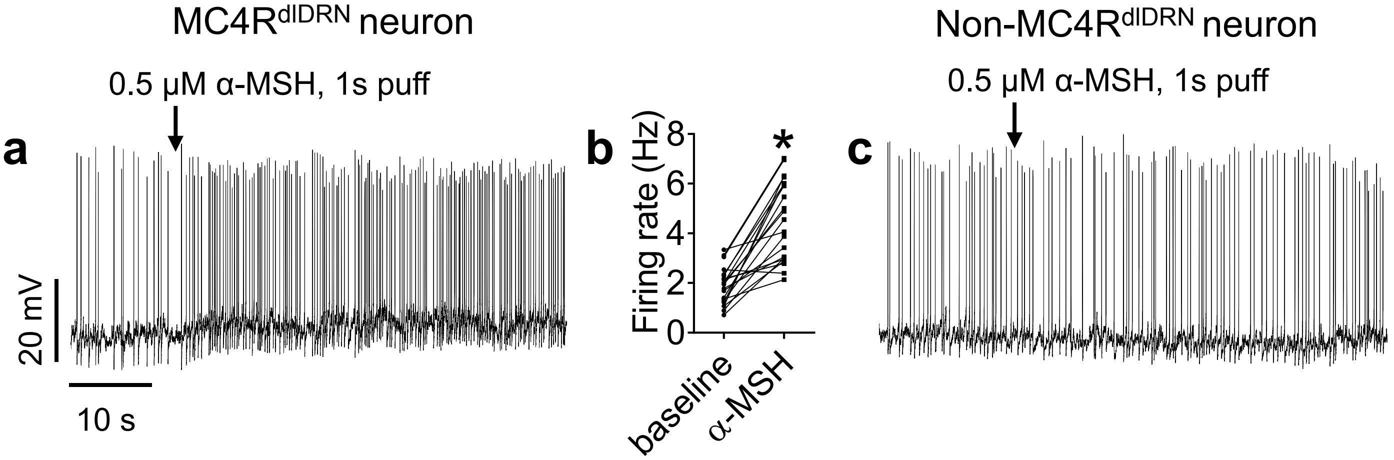

Supplementary Fig. 13. Effects of α-MSH on MC4R^{dlDRN} and non- MC4R^{dlDRN} neurons. a Representative trace from patch clamp recordings of MC4R^{dlDRN} neurons (**a**) and non- MC4R^{dlDRN} neurons (**c**) treated with α-MSH (1s puff) in mice with injection of *AAV2-DIO-ChR2-GFP* into the dlDRN of *Mc4r^{Cre}* mice. **b** Firing frequency of MC4R^{dlDRN} neurons as showed in **a** (n = 21 neurons from 5 mice; **P* < 0.0001; paired two-tailed t test). Error bars represent mean ± s.e.m.

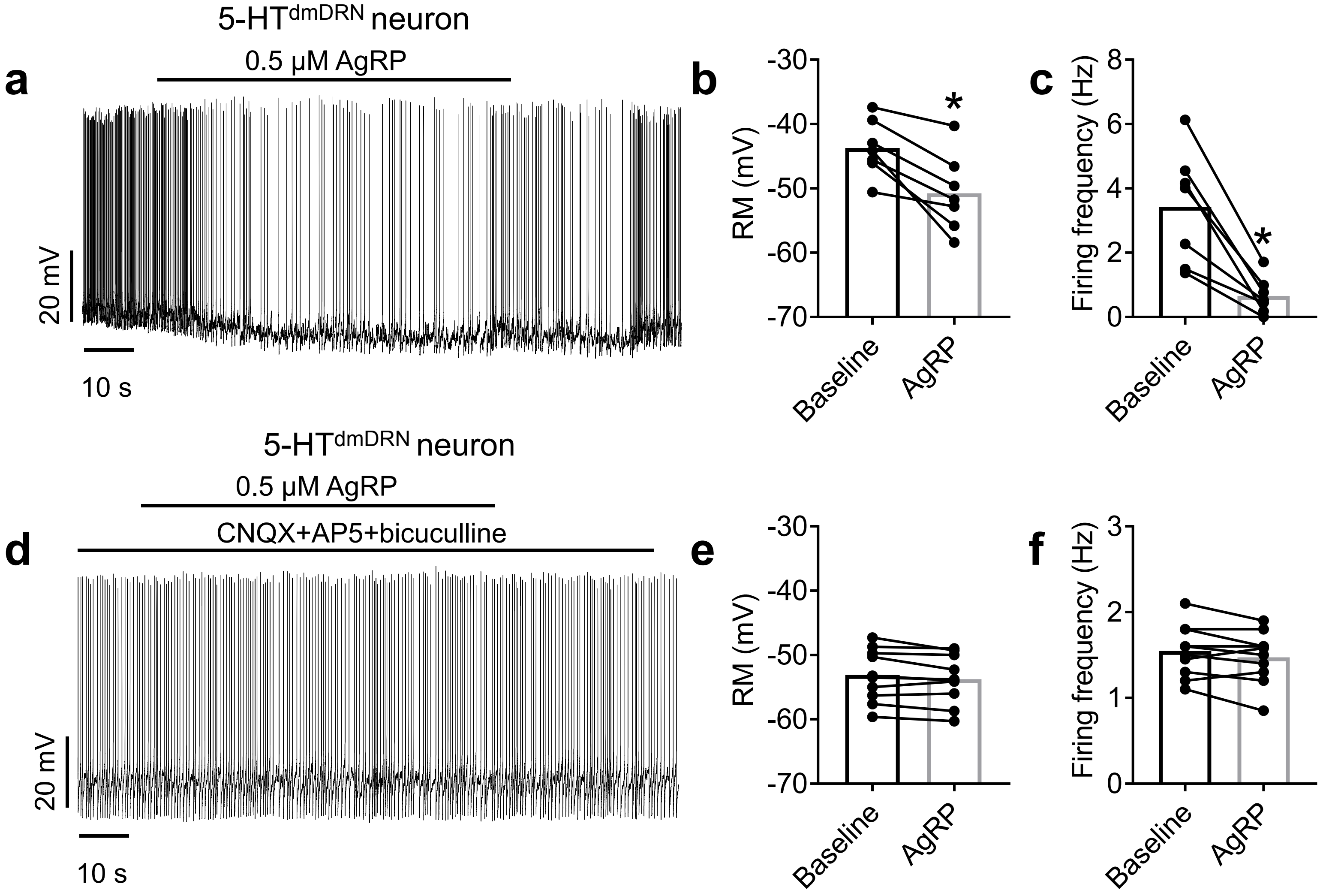

Supplementary Fig. 14. Effects of AgRP on 5-HT^{dmDRN} neurons. a Representative trace from patch clamp recordings of 5-HT^{dmDRN} neurons treated with AgRP (bath perfusion) in mice with injection of *AAV2-DIO-ChR2-GFP* into the dlDRN of *Mc4r^{Cre}* mice. The 5-HT^{dmDRN} neurons were identified by infusing lucifer yellow into cells after recording, followed by immunostaining with anti-Tph2. **b**-**c** RM potential (**b**) and firing frequency (**c**) of 5-HT^{dmDRN} neurons as showed in **a** (n = 7 neurons from 3 mice; for **b**, *P =0.0039, for **c**, *P =0.0017; paired two-tailed t test). **d** Representative trace from 5-HT^{dmDRN} neurons after application of CNQX, AP5 and bicuculline followed by AgRP in the mice described in **a**. **e**-**f** RM potential (**e**) and firing frequency (**f**) of 5-HT^{dmDRN} neurons as showed in **d** (n = 10 neurons from 3 mice). Error bars represent mean $\pm$ s.e.m.

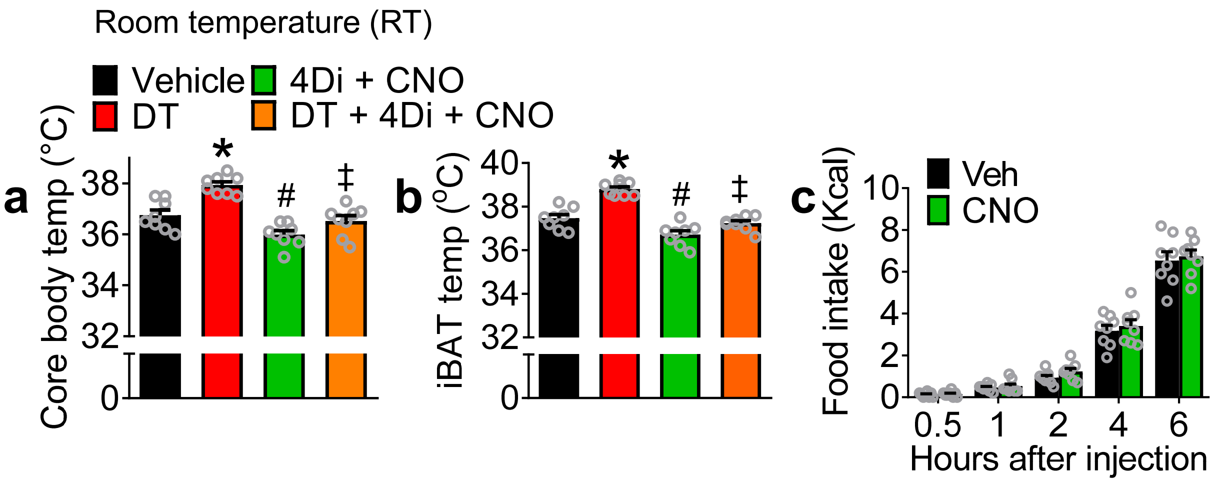

Supplementary Fig. 15. Suppression of MC4R^{dlDRN} neurons blunted the enhanced thermogenesis induced by ablation of AgRP$^{ARC \rightarrow dlDRN}$ neurons at room temperature. a-b Core body temperature (**a**) and iBAT temperature (**b**) at RT (23 °C) after i.p. treatment with CNO (1 mg/kg) in *Agrp$^{DTR/+}$::Mc4r^{Cre}* mice with *AAV2-DIO-hM4Di-mCherry* injection into the dlDRN. DT was injected into the dlDRN 14 days after viral injection. (n = 8 per group; $^{*}P$ was calculated between DT and vehicle, $^{\#}P$ was calculated between 4Di + CNO and vehicle; $^{\ddagger}P$ was calculated between DT + 4Di + CNO and DT; for **a**, F = 20.89, $^{*}P$ =0.0004, $^{\#}P$ =0.0275, $^{\ddagger}P$ <0.0001; for **b**, F = 36.65, $^{*}P$ =0.0001, $^{\#}P$ =0.0061, $^{\ddagger}P$ <0.0001; one-way ANOVA followed by Tukey post hoc test). **c** Accumulated food intake after i.p. treatment with CNO in the mice described in **a**, **b**. (n = 8 per group; all comparisons were made between Veh and CNO; P > 0.999 at hour 0.5, P = 0.999 at hour 1, P = 0.872 at hour 2, P = 0.948 at hour 4, P = 0.975 at hour 6; two-way ANOVA followed by Bonferroni post hoc test). Error bars represent mean ± s.e.m.

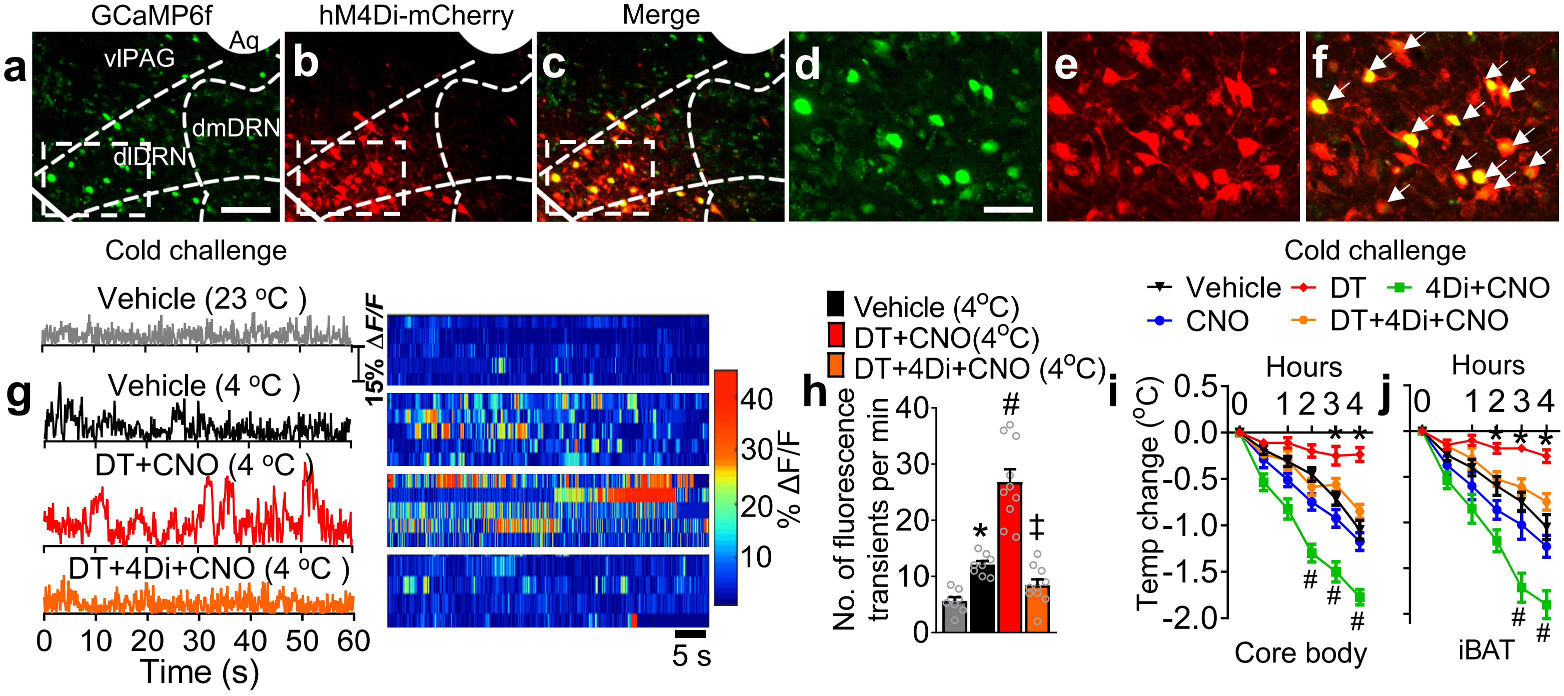

Supplementary Fig. 16. The MC4R^{dlDRN} neurons respond to thermogenesis during cold challenge. a-f *AAV5-FLEX-GCaMP6f* and *AAV2-DIO-hM4Di-mCherry* were injected into the dlDRN of *Agrp$^{DTR/+}$::Mc4r^{Cre}* mice to allow expression of GCaMP6f, a fast-acting calcium sensor, and hM4Di-mCherry within MC4R^{dlDRN} neurons. The colocolization of GCaMP6f and hM4Di-mCherry in the dlDRN with low magnification (**a-c**) and high magnification (**d-f**). Scale bar in **a** for **a-c**, 100 µm; Scale bar in **d** for **d-f**, 50 µm. **g-h** Representative traces of calcium signals (**g**, left), heating map (**g**, right), and fluorescence transients (**h**) of MC4R^{dlDRN} neurons under room temperature (RT, 23 °C) or a cold challenge (4 °C) after i.p. treatment with CNO (1 mg/kg) in *Agrp$^{DTR/+}$::Mc4r^{Cre}* mice injected by *AAV2-DIO-hM4Di-mCherry* and DT into the dlDRN 14 days apart. Scale bar in **g** are in rainbow scale (0 blue–50 red). (n = 8 in Vehicle (23 °C) and Vehicle (4 °C) group, n = 10 in DT+CNO (4 °C) and DT+4Di+CNO (4 °C) group; **P* was calculated between vehicle (23 °C) and vehicle (4 °C), $^{\#}P$ was calculated between vehicle (4 °C) and DT+CNO (4 °C); $^{‡}P$ was calculated between DT+CNO (4°C) and DT+4Di+CNO (4 °C); *F* = 36.72, **P* =0.0371, $^{\#}P$ <0.0001, $^{‡}P$ <0.0001; one-way ANOVA followed by Tukey post hoc test). **i-j** Core body temperature (**i**) and iBAT temperature (**j**) during a cold challenge in the mice described in **g** and **h**. (n = 8 per group; **P* was calculated between Vehicle and DT, $^{\#}P$ was calculated between DT+4Di+CNO and DT; for **i**, *F* = 98.65, **P* =0.0002 at hour 3, **P* <0.0001 at hour 4, $^{\#}P$ =0.0037 at hour 2, $^{\#}P$ =0.00393 at hour 3, $^{\#}P$ <0.0001 at hour 4; for **j**, *F* = 68.63, **P* =0.0319 at hour 2, **P* =0.0003 at hour 3, **P* <0.0001 at hour 4, $^{\#}P$ =0.0239 at hour 3, $^{\#}P$ =0.0037 at hour 4; two-way ANOVA followed by Bonferroni post hoc test). Error bars represent mean ± s.e.m.

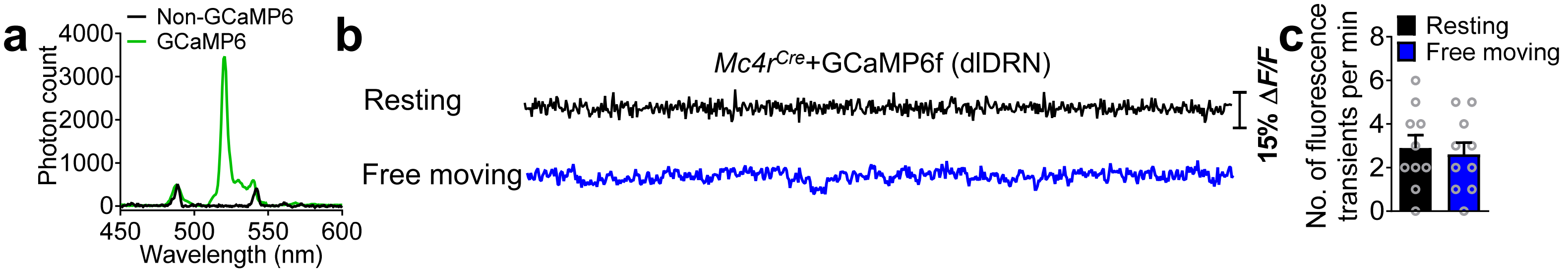

Supplementary Fig. 17. The changes in neural activity-dependent fluorescence from MC4R^{dlDRN} neurons are not sensitive to locomotion. **a** Fluorescence of GCaMP6f and brain autofluorescence measured from the dlDRN of *Mc4r*^{Cre} mice and WT mice, respectively. **b**-**c** Sample traces (**b**) and the number of fluorescence transients (**c**) detected in MC4R^{dlDRN} neurons of mice in resting and free-moving states. (n = 10 per group; P = 0.711 between Resting and Free moving group, unpaired two-tailed t test). Error bars represent mean ± s.e.m.

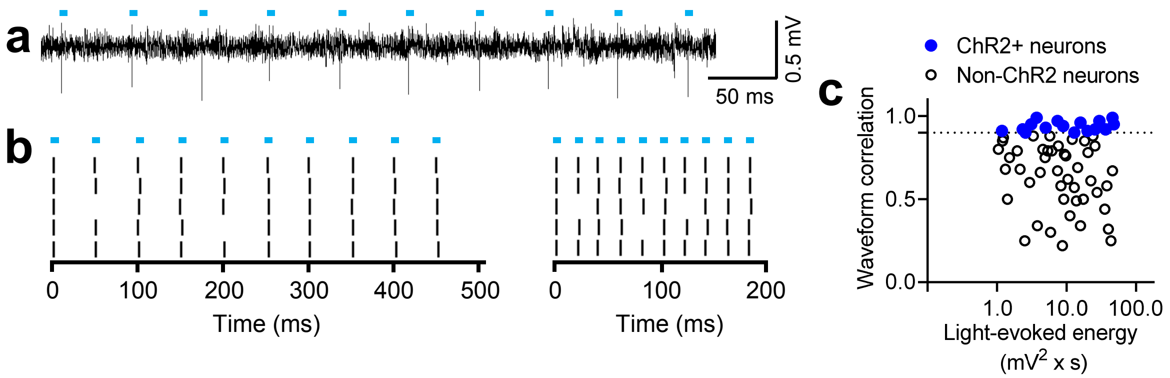

Supplementary Fig. 18. Identification of the ChR2-positive MC4R^{dlDRN} neurons. **a** Representative trace from 10 pulses during 20-Hz photostimulation. **b** Representative firing raster from this neuron at 20 Hz and 50 Hz stimulation. **c** Identification of MC4R^{dlDRN} neurons by evoked responses during photostimulation. Identified MC4R^{dlDRN} neurons are indicated by filled circles.

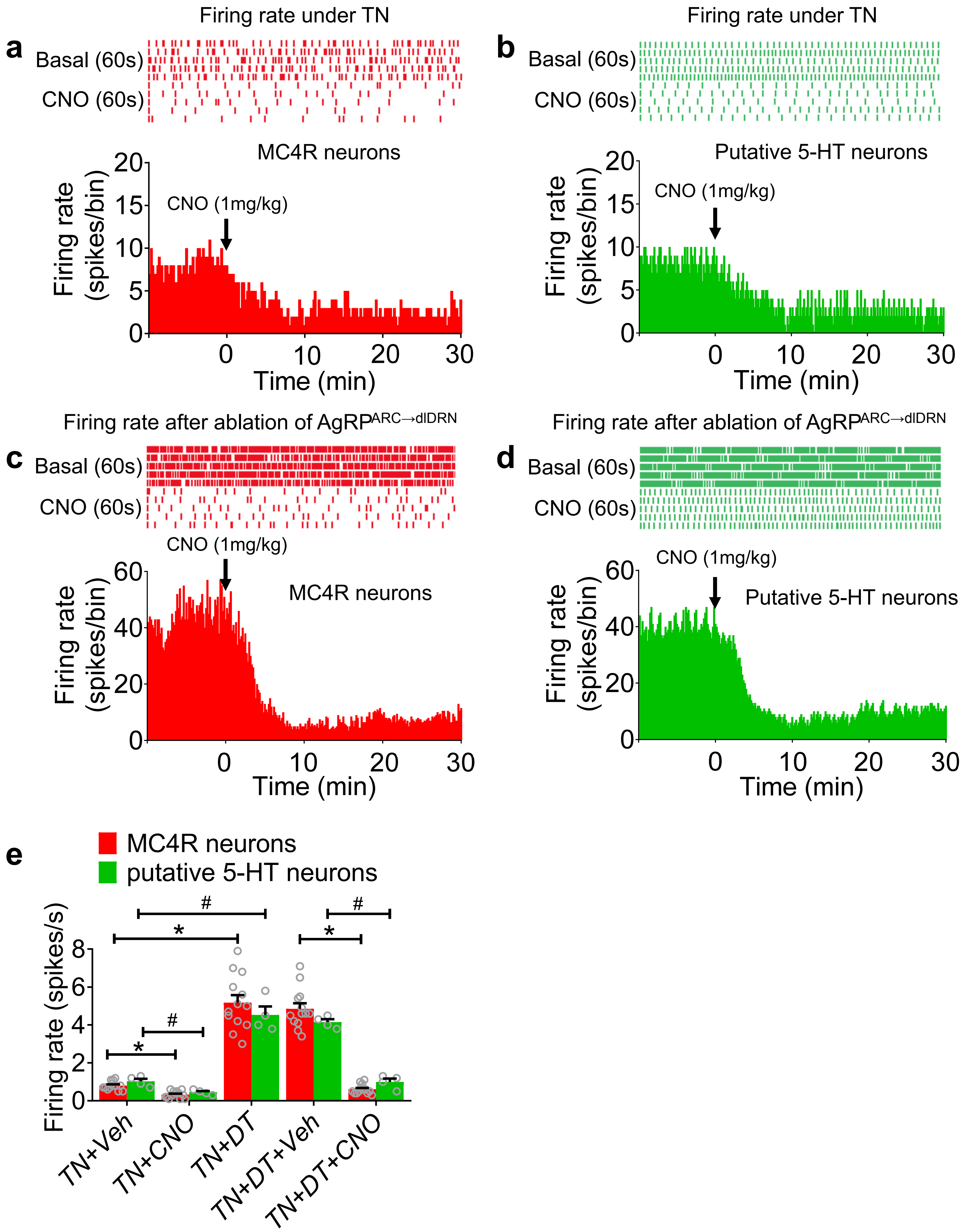
a
Firing rate under TN
Basal (60s)
CNO (60s)
MC4R neurons
CNO (1mg/kg)
Firing rate (spikes/bin)
Time (min)
b
Firing rate under TN
Basal (60s)
CNO (60s)
Putative 5-HT neurons
CNO (1mg/kg)
Firing rate (spikes/bin)
Time (min)
c
Firing rate after ablation of AgRP$^{ARC\rightarrow dlDRN}$
Basal (60s)
CNO (60s)
CNO (1mg/kg)
MC4R neurons
Firing rate (spikes/bin)
Time (min)
d
Firing rate after ablation of AgRP$^{ARC\rightarrow dlDRN}$
Basal (60s)
CNO (60s)
CNO (1mg/kg)
Putative 5-HT neurons
Firing rate (spikes/bin)
Time (min)
e
MC4R neurons
putative 5-HT neurons
Firing rate (spikes/s)
TN+Veh
TN+CNO
TN+DT
TN+DT+Veh
TN+DT+CNO

Supplementary Fig. 19. The MC4R^{dlDRN} and 5-HT^{dmDRN} neurons are chemogenetically regulated by CNO under TN. **a**-**b** Raster plots (above) and histograms (below) of firing rate of MC4R^{dlDRN} neurons (**a**) and putative 5-HT^{dmDRN} neurons (**b**) under TN conditions before and after CNO injection in the *Agrp$^{DTR/+}$::Mc4r^{Cre}* mice after injection of *AAV2-DIO-ChR2-GFP* and *AAV2-DIO-hM4Di-mCherry* into the dlDRN with *in vivo* optrode recording. **c**-**d** Raster plots and histograms of firing rates of MC4R^{dlDRN} neurons (**c**) and 5-HT^{dmDRN} neurons (**d**) under TN conditions before and after CNO injection, while AgRP$^{ARC\rightarrow dlDRN}$ neurons were ablated by DT. **e** Firing rates in MC4R^{dlDRN} neurons and putative 5-HT^{dmDRN} neurons before and after vehicle or CNO, with or without ablation of AgRP$^{ARC\rightarrow dlDRN}$ neurons. (n = 13 for MC4R^{dlDRN} neurons, n = 4 for 5-HT^{dmDRN} neurons; F = 101.8, for MC4R^{dlDRN} neurons, *P <0.0001 TN+Veh vs TN+CNO, *P < 0.0001 TN+Veh vs TN+DT, *P < 0.0001 TN+DT+Veh vs TN+DT+CNO; for 5-HT^{dmDRN} neurons, $^{\#}P$ < 0.0001 TN+Veh vs TN+CNO, $^{\#}P$ < 0.0001 TN+Veh vs TN+DT, $^{\#}P$ < 0.0001 TN+DT+Veh vs TN+DT+CNO; two-way ANOVA followed by Bonferroni post hoc test). Error bars represent mean ± s.e.m.

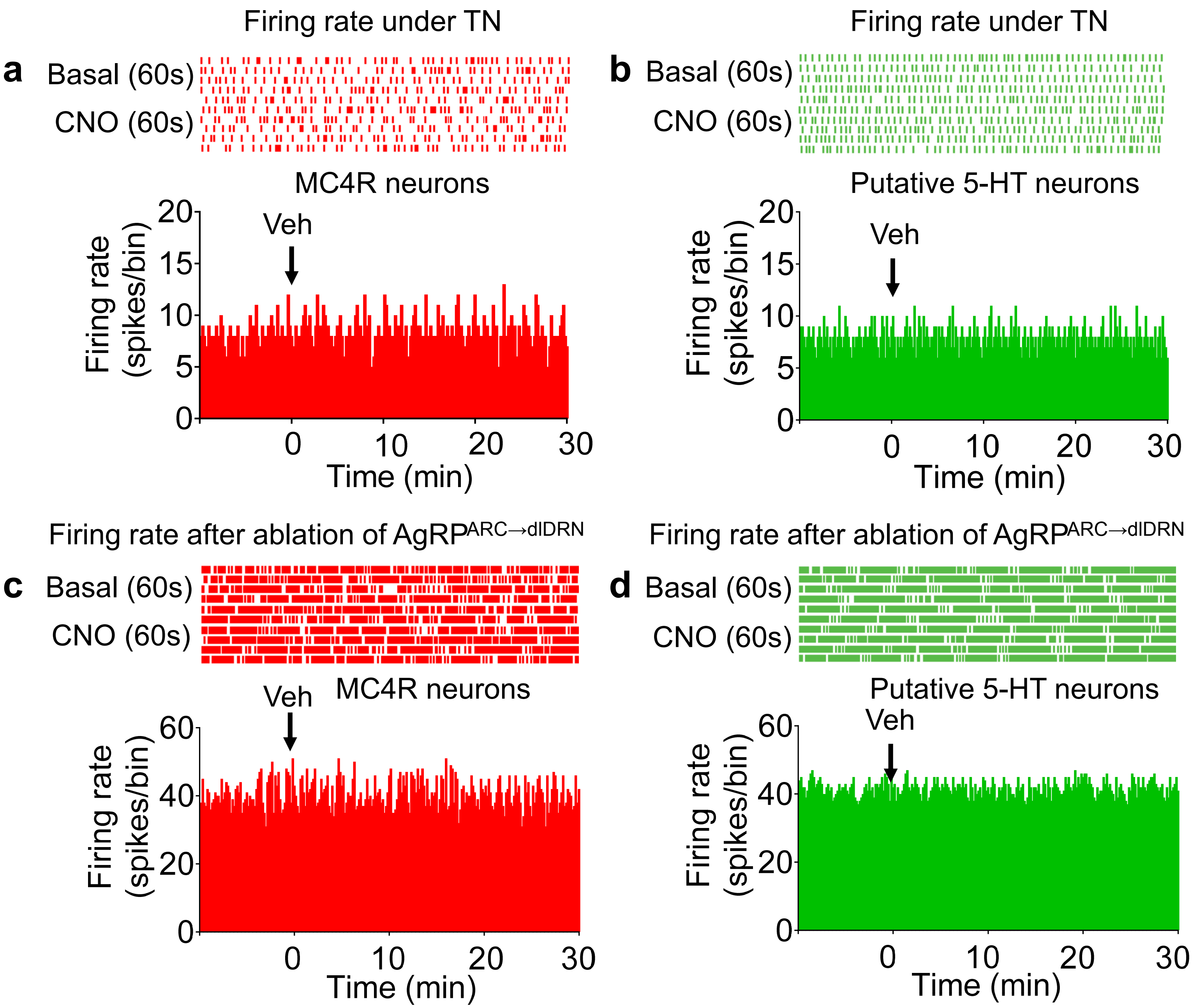

Supplementary Fig. 20. The effects of vehicle on the MC4R^{dlDRN} and 5-HT^{dmDRN} neurons during opto-tetrode recording. a-b Raster plots (above) and histograms (below) of firing rate of MC4R^{dlDRN} neurons (**a**) and putative 5-HT^{dmDRN} neurons (**b**) under TN conditions before and after vehicle injection. **c-d** Raster plots and histograms of firing rates of MC4R^{dlDRN} neurons (**c**) and putative 5-HT^{dmDRN} neurons (**d**) under TN conditions before and after vehicle injection, while AgRP$^{ARC\rightarrow dlDRN}$ neurons were ablated by DT.

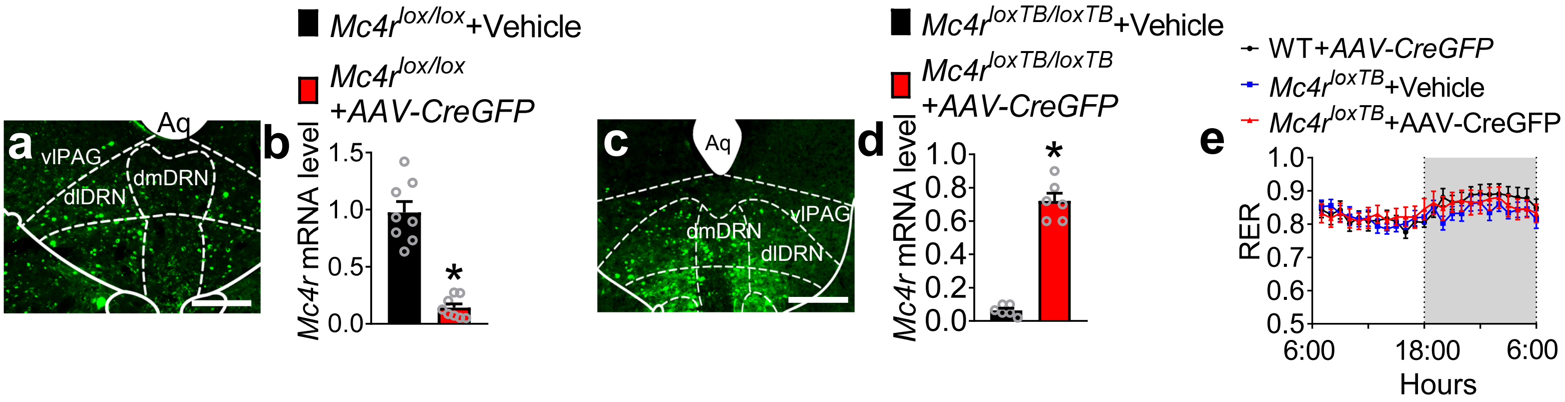

Supplementary Fig. 21. Genetic manipulation of MC4R signaling in the dlDRN. **a** Image showing viral transduction upon injection of *AAV2-CreGFP* into the dlDRN of *Mc4r*$^{lox/lox}$ mice. Scale bar, 200 μm. **b** Quantification of *Mc4r* transcription levels in the dlDRN by qPCR 14 days after injection of *AAV2-CreGFP* or vehicle (*AAV2-GFP*) into the dlDRN of mice described in **a**. (n = 8 per group; F = 8.292, *P < 0.0001; unpaired two-tailed t test). **c** Image showing viral transduction upon injection of *AAV2-CreGFP* in the dlDRN of *Mc4r*$^{loxTB/loxTB}$ mice. Scale bar, 200 μm. **d** Quantification of *Mc4r* transcription levels in the dlDRN by qPCR 14 days after viral injection of *AAV2-CreGFP* or vehicle (*AAV2-GFP*) into the dlDRN of mice described in **c**. (n = 6 per group; F = 8.070, *P < 0.0001, unpaired two-tailed t test). **e** RER measured in the mice described in **c**. (n = 8 per group). Error bars represent mean ± s.e.m.

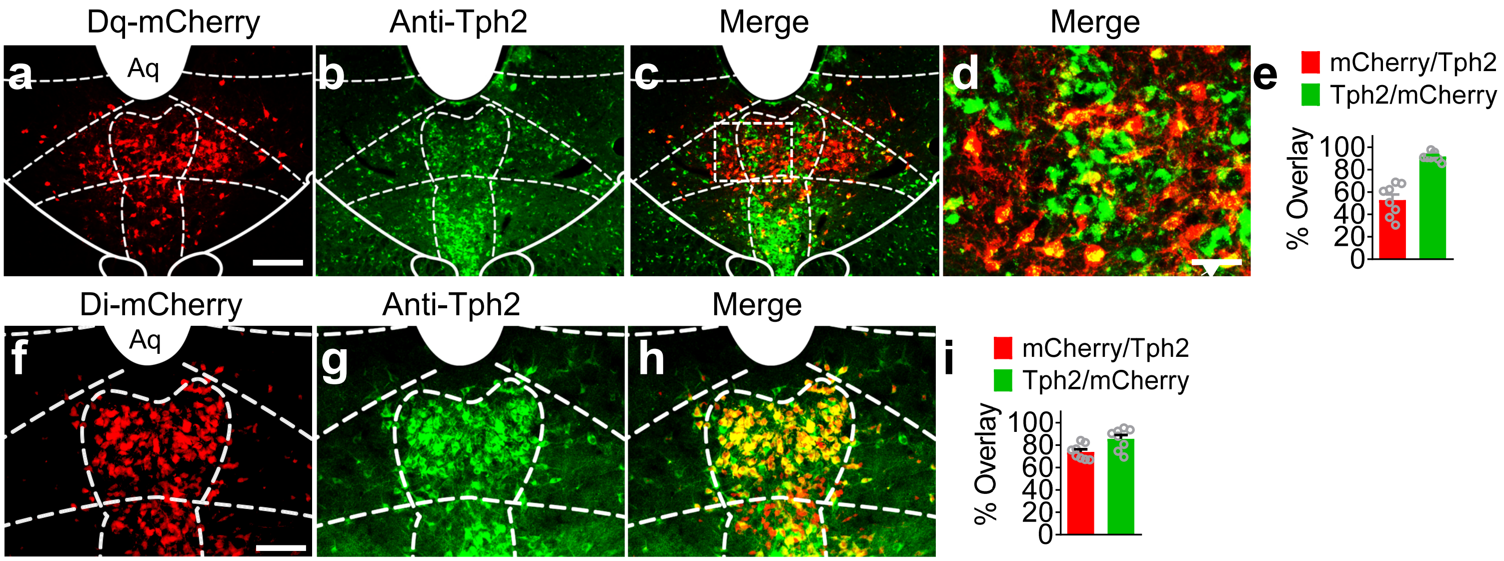

Supplementary Fig. 22. Efficacy of viral transduction in the 5-HT^{dmDRN} neurons. a-d Immunostaining image of the DRN in *Pet1^{Cre}* mice with *AAV2-DIO-hM3Dq-mCherry* injected into the dmDRN (anti-Tph2, Green; Dq-mCherry, Red) with low magnification (**a-c**) and high magnification (**d**). Scale bar in **a** for **a-c**, 150 μm; Scale bar in **d**, 50 μm. **e** Quantification of overlap of Dq-mCherry and Tph2. (n = 8 per group). **f-h** Immunostaining image of the DRN in *Pet1^{Cre}* mice injected with *AAV2-DIO-hM4Di-mCherry* into the dmDRN (anti-Tph2, Green; Di-mCherry, Red). Scale bar in **f** for **f-h**, 100 μm. **i** Quantification of overlap of Di-mCherry and Tph2. (n = 8 per group). Error bars represent mean ± s.e.m.

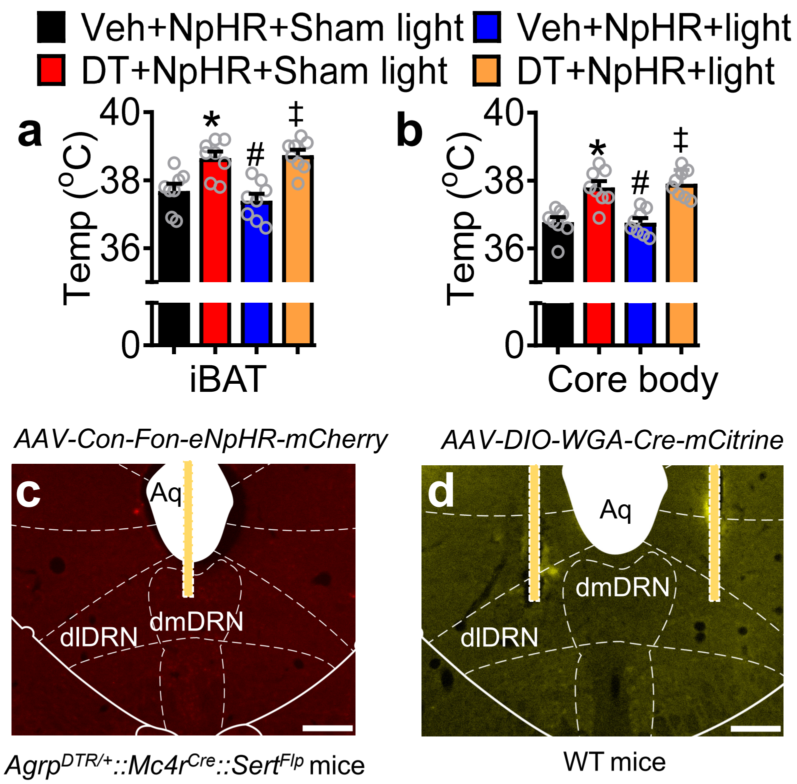

Supplementary Fig. 23. The effects of single viral injection on thermogenesis in absence of expression of NpHR in the dmDRN. a-b The iBAT temperature (**a**) and core body temperature (**b**) 1 hour after photoinhibition in the DRN in the *Agrp*$^{DTR/+}$*::Mc4r*^{Cre}*::Sert*^{Flp} mice with injection of *AAV9-Con-Fon-eNpHR-mCherry* into the dmDRN. DT was injected into dlDRN 4 weeks after virus injection with an optical fiber inserted into the dmDRN. The iBAT and core body temperature were measured 7 days after DT injection. (n = 8 per group; **P* was calculated between Veh+NpHR+Sham light and DT+NpHR+Sham, $^{\#}P$ was calculated between Veh+NpHR+Sham light and Veh+NpHR+light, $^{‡}P$ was calculated between DT+NpHR+Sham light vs DT+NpHR+light; for **a**: F = 12.53, $^{*}P$ =0.0068, $^{\#}P$ =0.7126, $^{‡}P$ =0.9879; for **b**: F = 17.20, $^{*}P$ =0.0003, $^{\#}P$ >0.9999, $^{‡}P$ =0.9526; One-way ANOVA followed by Tukey post hoc test). **c** No positive fluorescence detected in the DRN. Scale bar, 150 μm. **d** No positive fluorescence detected in the DRN in the WT mice with injection of *AAV-DIO-WGA-Cre-mCitrine* into the dlDRN. Scale bar, 150 μm. Error bars represent mean ± s.e.m.

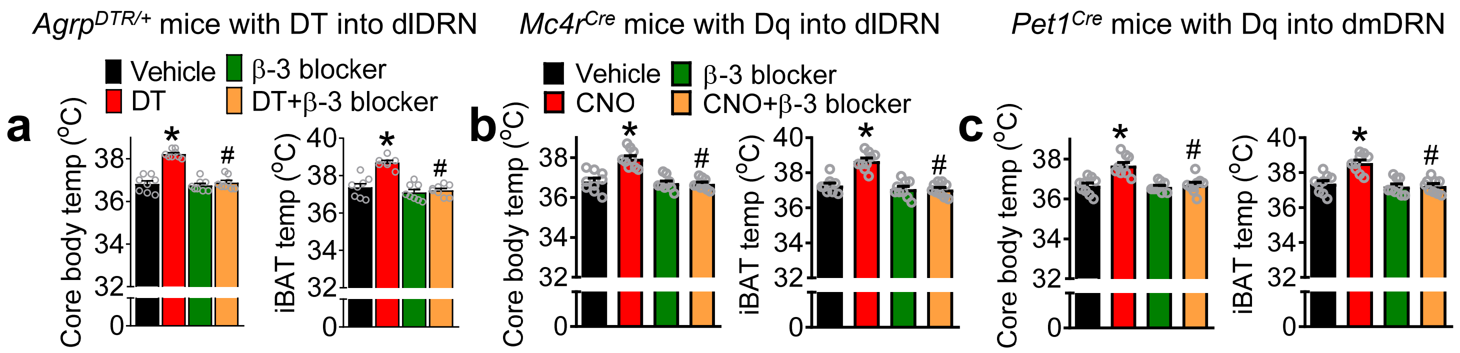

Supplementary Fig. 24. The effects of β-3 blockers on thermogenesis in the manipulation of MC4R$^{\mathbf{dlDRN}}$ and 5-HT$^{\mathbf{dmDRN}}$ neurons. a Core body temperature and iBAT temperature in WT or *Agrp$^{DTR/+}$:: Npy^{GFP}* mice with bilateral injection of DT (0.4 ng/side) into the dlDRN with a pretreatment of SR59230A (a selective β-3AR blocker, 2 mg/kg/day, s.c. for 4 days). (n = 8 per group; **P* was calculated between DT and Vehicle, $^{\#}P$ was calculated between DT and DT+β-3 blocker; F = 44.22 (left), F = 23.31 (right), $^{*}P < 0.0001$, $^{\#}P < 0.0001$; one-way ANOVA followed by Tukey post hoc test). **b** Core body temperature and iBAT temperature in *Mc4r^{Cre}* mice injected by *AAV2-DIO-hM3Dq-mCherry* into dlDRN with a pretreatment of SR59230A. (n = 9 per group; **P* was calculated between CNO and Vehicle, $^{\#}P$ was calculated between CNO and CNO+β-3 blocker; F = 20.09 (left), F = 27.06 (right), $^{*}P < 0.0001$, $^{\#}P < 0.0001$; one-way ANOVA followed by Tukey post hoc test). **c** Core body temperature and iBAT temperature in *Pet1^{Cre}* mice injected with *AAV2-DIO-hM3Dq-mCherry* into dmDRN with a pretreatment of SR59230A. (n = 9 per group; **P* was calculated between CNO and Vehicle, $^{\#}P$ was calculated between CNO and CNO+β-3 blocker; F = 14.68 (left), F = 15.89 (right), $^{*}P < 0.0001$, $^{\#}P < 0.0001$; one-way ANOVA followed by Tukey post hoc test). Error bars represent mean ± s.e.m.

	Supplementary Table 1. Quantitative PCR primer sequences	
Gene Symbol	Sequences (5’ to 3’)	
	Forward	Reverse
Mc4r	GCCTGCATTACCGGTCGATGC	CCCTGATCCTGGCAATTTCGGC
Tph2	CATTCCTCGCACAATTCCAGTCG	AGTCTACATCCATCCCAACTGCTG
Ucp1	AGCCACCACAGAAAGCTTGTCAAC	ACAGCTTGGTACGCTTGGGTACTG
Ppargc1a	CCTGCATGAGTGTGTGCTCT	CTCAGAGTCCTGGTTGCACA
Cidea	TCAGACCTTAAGGGACAACACGCA	TTCTTTGGTTGCTTGCAGACTGGG
Cited1	CCGTACCTCAGCTCCTGTG	AGCTGGGCCTGTTGGTCT
Dio2	CAGTGTGGTGCACGTCTCCAATC	TGAACCAAAGTTGACCACCAG
Cox7a1	CAGCGTCATGGTCAGTCTGT	AGAAAACCGTGTGGCAGAGA
Car4	TACGTGGCCCCCTCTACTG	GCTGATTCTCCTTACAGGCTCC
Fgf21	AGATGGAGCTCTCTATGGATCG	GGGCTTCAGACTGGTACACAT
Eva1	GTCCCAACCAGACCATCAAC	CTCCATCTTGCTCTGGAAGC
Scr	TAATACGACTCACTATAGGG	CTGGAATAGCTCAGAGGC
Gapdh	GTGGAGTCATACTGGAACATGTAG	AATGGTGAAGGTCGGTGTG

Supplementary Table 1. Primer sequences for qRT-PCR. The qRT-PCR was performed using TaqMan gene expression assay for *Mc4r* , *Tph2* , *Ucp1* , *Ppargc1a* , *Cidea* , *Cited1* or using SYBR Green system for *Gapdh* , *Dio2* , *Cox7a1* , *Car4* , *Fgf21* , *Eva1* , and *Scr* .
