# Supplementary material for: Deciphering an AgRP-serotoninergic neural circuit in distinct control of energy metabolism from feeding
Source: Nat Commun. 2021 Jun 10;12:3525. doi: 10.1038/s41467-021-23846-x (PMC8192783; doi:10.1038/s41467-021-23846-x)
Supplement: Supplementary file 3 — Reporting Summary [file 41467_2021_23846_MOESM3_ESM.pdf]

nature research

Corresponding author(s): Qi Wu

Last updated by author(s): Apr 4, 2021

Reporting Summary

Nature Research wishes to improve the reproducibility of the work that we publish. This form provides structure for consistency and transparency in reporting. For further information on Nature Research policies, see our Editorial Policies and the Editorial Policy Checklist.

Statistics

For all statistical analyses, confirm that the following items are present in the figure legend, table legend, main text, or Methods section.

n/a	Confirmed	
☐	☒	The exact sample size (n) for each experimental group/condition, given as a discrete number and unit of measurement
☐	☒	A statement on whether measurements were taken from distinct samples or whether the same sample was measured repeatedly
☐	☒	The statistical test(s) used AND whether they are one- or two-sided<br>*Only common tests should be described solely by name; describe more complex techniques in the Methods section.*
☒	☐	A description of all covariates tested
☐	☒	A description of any assumptions or corrections, such as tests of normality and adjustment for multiple comparisons
☐	☒	A full description of the statistical parameters including central tendency (e.g. means) or other basic estimates (e.g. regression coefficient) AND variation (e.g. standard deviation) or associated estimates of uncertainty (e.g. confidence intervals)
☐	☒	For null hypothesis testing, the test statistic (e.g. F, t, r) with confidence intervals, effect sizes, degrees of freedom and P value noted<br>*Give P values as exact values whenever suitable.*
☒	☐	For Bayesian analysis, information on the choice of priors and Markov chain Monte Carlo settings
☒	☐	For hierarchical and complex designs, identification of the appropriate level for tests and full reporting of outcomes
☒	☐	Estimates of effect sizes (e.g. Cohen's d, Pearson's r), indicating how they were calculated

Our web collection on statistics for biologists contains articles on many of the points above.

Software and code

Policy information about availability of computer code

Data collection	The CLAMS data were collected from Oxymax Software version 5.12. The LC-MS data were processed using Thermo Scientific Proteome Discoverer software version 1.4. The in vitro electrophysiological data were collected and analyzed offline with pClamp software version 10.3. In vivo fiber photometry data were acquired by OceanView software version 1.2. The in vivo tetrode recording data were collected using Cheetah software version 5.6.3. Seahorse data were collected and analyzed using Seahorse Wave software version 2.4. The fluorescent images were captured using AxioVision software version 4.9.1
Data analysis	Fluorescent images were analyzed using ImageJ software version 1.49. The in vivo fiber photometry data were analyzed using MATLAB R2015a. The in vivo tetrode recording data were sorted using Plexon Offline Sorter software version 4.4.1 and analyzed using NeuroExplorer version 5.033. All statistical analyses were performed by Graphpad Prism software version 7.00.

For manuscripts utilizing custom algorithms or software that are central to the research but not yet described in published literature, software must be made available to editors and reviewers. We strongly encourage code deposition in a community repository (e.g. GitHub). See the Nature Research guidelines for submitting code & software for further information.

Data

Policy information about availability of data

All manuscripts must include a data availability statement. This statement should provide the following information, where applicable:

- Accession codes, unique identifiers, or web links for publicly available datasets
- A list of figures that have associated raw data
- A description of any restrictions on data availability

We state that all figures in this manuscript have associated raw data.

Field-specific reporting

Please select the one below that is the best fit for your research. If you are not sure, read the appropriate sections before making your selection.

☒ Life sciences ☐ Behavioural & social sciences ☐ Ecological, evolutionary & environmental sciences

For a reference copy of the document with all sections, see nature.com/documents/nr-reporting-summary-flat.pdf

Life sciences study design

All studies must disclose on these points even when the disclosure is negative.

Sample size	Sample size was determined by power analysis in order to achieve statistical and scientific significance.
Data exclusions	No data were excluded for this manuscript.
Replication	Experimental findings were reliably repeated in 2-3 times independent experiments.
Randomization	Allocation of animals in each experiment was randomized.
Blinding	For all experiments in this studies, subjects were randomly assigned to the various experimental conditions. However, the experimenters were not blinded to group allocation during data acquisition since the same experimenter had to administer the various treatments.

Reporting for specific materials, systems and methods

We require information from authors about some types of materials, experimental systems and methods used in many studies. Here, indicate whether each material, system or method listed is relevant to your study. If you are not sure if a list item applies to your research, read the appropriate section before selecting a response.

Materials & experimental systems

n/a	Involved in the study	
☐	☒	Antibodies
☒	☐	Eukaryotic cell lines
☒	☐	Palaeontology and archaeology
☐	☒	Animals and other organisms
☒	☐	Human research participants
☒	☐	Clinical data
☒	☐	Dual use research of concern

Methods

n/a	Involved in the study	
☒	☐	ChIP-seq
☒	☐	Flow cytometry
☒	☐	MRI-based neuroimaging

Antibodies

Antibodies used	goat anti-AgRP (sc-18634, Santa Cruz Biotech, Dallas, TX), rabbit anti-Tph2 (ABN60, EMD Millipore, Burlington, MA), chicken anti-GFP (A10262, Invitrogen, Waltham, MA), rabbit Anti-Iba1 (PA5-27436, Thermo Fisher Scientific, Waltham, MA), Alex Fluor 488-conjugated donkey anti-rabbit secondary antibody (711-545-152, Jackson Immunolab, West Grove, PA), Alex Fluor 488-conjugated goat anti-chicken secondary antibody (A-11039, Thermo Fisher Scientific, Waltham, MA), Alex Fluor Cy3-conjugated donkey anti-rabbit secondary antibody (711-585-152, Jackson Immunolab, West Grove, PA), Alex Fluor Cy3-conjugated donkey anti-goat secondary antibody (705-585-147, Jackson Immunolab, West Grove, PA), Alex Fluor Cy5-conjugated donkey anti-rabbit secondary antibody (711-175-152, Jackson Immunolab, West Grove, PA)
Validation	All antibodies come with vendor's validation statements on the websites including detailed validation information and references.<br><br>For the AgRP antibody, AGRP (M-20) is an affinity purified goat polyclonal antibody raised against a peptide mapping within an internal region of AGRP of mouse origin. AGRP (M-20) is recommended for detection of AGRP of mouse and rat origin by Western Blotting (starting dilution 1:200, dilution range 1:100- 1:1000), immunofluorescence (starting dilution 1:50, dilution range 1:50- 1:500) and solid phase ELISA (starting dilution 1:30, dilution range 1:30- 1:3000). Select product citations: 1. Moraes, J.C., et al. 2009. High-fat diet induces apoptosis of hypothalamic neurons. PLoS ONE 4: e5045. 2. Kim, S.J., et al. 2009. Rutecarpine ameliorates bodyweight gain through the inhibition of orexigenic neuropeptides NPY and AgRP in mice. Biochem. Biophys. Res. Commun. 389: 437-442. 3. Colldén, G., et al. 2010. P2X2 purinoreceptor protein in hypothalamic neurons associated with the regulation of food intake. Neuroscience 171: 62-78. 4. Sasaki, T., et al. 2010. Induction of hypothalamic Sirt1 leads to cessation of feeding via agouti-related peptide. Endocrinology 151: 2556-2566. 5. Razolli, D.S., et al. 2012. Hypothalamic action of glutamate leads to body mass reduction through a mechanism partially dependent on JAK2. J. Cell. Biochem. 113: 1182-1189.<br><br>For the Tph2 antibody, species reactivity: mouse, rat, human. Application: Anti-Tryptophan hydroxylase 2 Antibody detects level of Tryptophan hydroxylase 2 & has been published & validated for use in WB, IH(P). Select product citations: 1. Mammalian-specific sequences in pou3f2 contribute to maternal behavior. Nasu, M; Yada, S; Igarashi, A; Sutoo, D; Akiyama, K; Ito, M; Yoshida, N; Ueda, S

Genome biology and evolution 6 1145-56 2014. 2. Differential postpartum sensitivity to the anxiety-modulating effects of offspring contact is associated with innate anxiety and brainstem levels of dopamine beta-hydroxylase in female laboratory rats. Ragan, CM; Lonstein, JS Neuroscience 256 433-44 2014.

For the GFP antibody, species reactivity: fruit fly, mouse, tag. Antibody specificity was demonstrated by detection of different targets fused to GFP tag in transiently transfected lysates tested. Relative detection of GFP tag was observed across different proteins fused with GFP in H3-GFP (Lane 3-5) and p65-GFP (Lane 6). GFP-variant, YFP is also being detected in His-p65-YFP lysate (Lane 7), using Anti-GFP Polyclonal Antibody (Product # A10262) in Western Blot. Select product citations: 1. Expression of the novel maternal centrosome assembly factor Wdr8 is required for vertebrate embryonic mitoses. Nature communications. 2017. 2. Antagonistic negative and positive neurons of the basolateral amygdala. Nature neuroscience. 2016.

For Iba1 antibody, species reactivity: mouse, rat, human. This Antibody was verified by Knockout to ensure that the antibody binds to the antigen stated. Antibody specificity was demonstrated by CRISPR-Cas9 mediated knockout of target protein. A loss of signal was observed for target protein in IBA1 KO cell line compared to control cell line using Anti-IBA1 Polyclonal Antibody (Product # PA5-27436). Select product citations: 1. Human Inner Ear Immune Activity: A Super-Resolution Immunohistochemistry Study. Frontiers in neurology 2020. 2. The Transient Receptor Potential Melastatin 2 (TRPM2) Channel Contributes to β-Amyloid Oligomer-Related Neurotoxicity and Memory Impairment. The Journal of neuroscience. 2015.

Animals and other organisms

Policy information about studies involving animals; ARRIVE guidelines recommended for reporting animal research

Laboratory animals	All animals were C57BL6 mouse strains with both male and female have been tested. Mice used for experiments were 8-16 weeks old; and kept in a temperature (22°C) and humidity (40-60%)-controlled rooms, in a 12/12 hr light/dark cycle, with lights on from 6:00 AM–6:00 PM. Agrp-DTR mice, Npy-GFP mice, Agrp-Cre mice, Mc4r-Cre mice, Sert-Flp mice, Mc4r-loxTB mice, Mc4r-lox/lox mice, Pet1-Cre mice, Ai32 mice, Ai14 (or Rosa26tdTomato) mice were used in studies.
Wild animals	The study did not involve wild animals.
Field-collected samples	The study did not involve samples collected from field.
Ethics oversight	This study followed the highest ethical practices and compliance standards according the policy of Baylor College of Medicine. All animal care and experimental procedures were approved by the Institutional Animal Care and Use Committees at Baylor College of Medicine and were performed in accordance with the guidelines described in the NIH guide for the care and use of laboratory animals.

Note that full information on the approval of the study protocol must also be provided in the manuscript.
